# Asymmetric synthesis of quaternary aryl amino acid derivatives via a three-component aryne coupling reaction

**DOI:** 10.3762/bjoc.7.185

**Published:** 2011-11-25

**Authors:** Elizabeth P Jones, Peter Jones, Andrew J P White, Anthony G M Barrett

**Affiliations:** 1 Department of Chemistry, Imperial College London, London, SW7 2AZ, England; 2 Worldwide Medicinal Chemistry, Pfizer Limited, Ramsgate Road, Sandwich, Kent, CT13 9NJ, England

**Keywords:** aryl amino acids, arynes, asymmetric, multicomponent, quaternary

## Abstract

A method was developed for the synthesis of α-alkyl, α-aryl-bislactim ethers in good to excellent yields and high diastereoselectivities, consisting of a facile one-pot procedure in which the aryl group is introduced by means of a nucleophilic addition to benzyne and the alkyl group by alkylation of a resultant benzylic anion. Hydrolysis of the sterically less hindered adducts gave the corresponding quaternary amino acids with no racemization, whereas hydrolytic ring opening gave the corresponding valine dipeptides from bulkier bislactims.

## Introduction

**Scheme 1:**
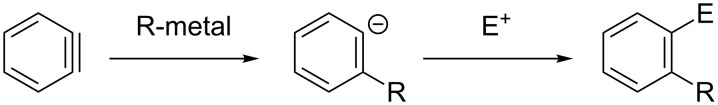
3-Component coupling reactions of arynes. E^+^ = electrophile.

Arynes are exceptionally versatile reactive intermediates in organic synthesis. Not only are they able to participate in cycloaddition reactions, they also readily undergo addition reactions with nucleophiles, and the resultant aryl anions may be protonated or undergo alternative transformations [1–6]. We are particularly interested in the use of arynes in multicomponent coupling reactions, in which the resultant aryl carbanion following nucleophilic attack is allowed to react with a further electrophile, providing *ortho*-disubstituted, functionalized aromatic products in a one-pot procedure [7–9]. In particular, we have focused on the addition of carbon-based nucleophiles in such systems (Scheme 1) and have applied this methodology to the syntheses of natural products ent-clavilactone B and dehydroaltenuene B [10–11].

**Scheme 2:**
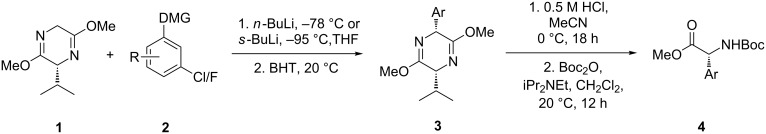
Aryne mediated α-arylation of amino acids. DMG = directed metallation group. BHT = 2,6-di-*tert*-butyl-4-methylphenol.

Recently, we reported the diastereoselective addition of Schöllkopf’s bislactim ether **1** [12] to substituted arynes **2**, which, following hydrolysis of the *syn*-adducts **3**, provided α-aryl amino acids **4** with moderate to high enantioselectivities (Scheme 2) [13].

**Scheme 3:**
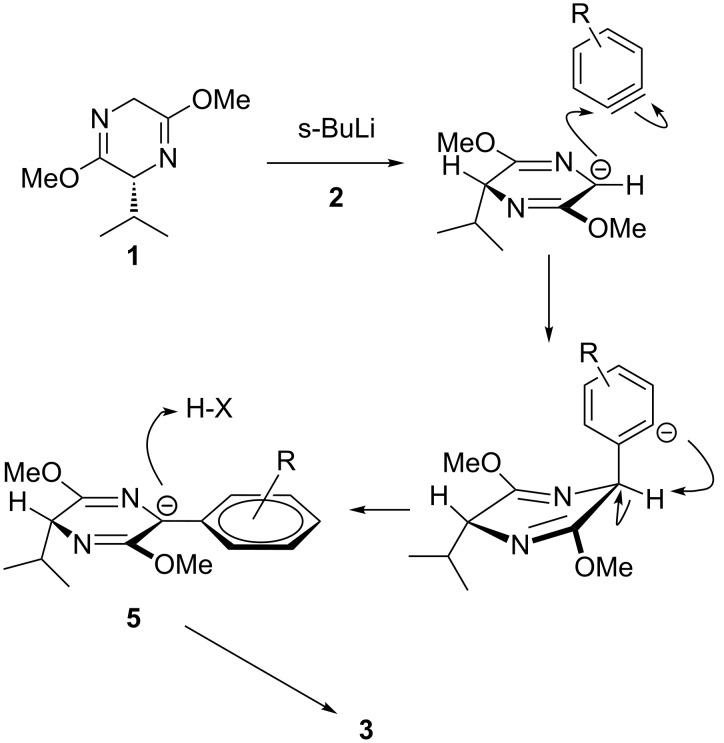
Proposed mechanism of α-arylation.

The arynes were produced by a directed *ortho*-metallation [14] and an elimination sequence, and after attack of **1** the ensuing carbanion **5** was not aryl as expected but instead benzylic, due to an inter- or intramolecular proton transfer. Kinetic protonation of this anion on the face opposite to the isopropyl group accounted for the observed diastereoselectivity of the reaction (Scheme 3).

**Scheme 4:**
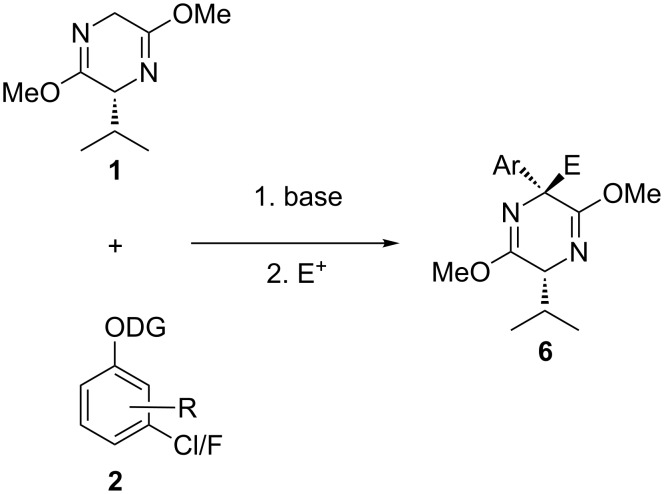
Proposed extension of the methodology to synthesize quaternary adducts.

To extend this methodology to a multicomponent system, we considered that quenching the key aza-enolate intermediate with an electrophile, rather than a proton source, should provide quaternary Schöllkopf adducts **6** (Scheme 4).

The potential utility of chiral α-quaternary α-aryl amino acids in medicinal chemistry is diverse. Incorporation of such units into peptidomimetics, not only affects lipophilicity, but also the secondary structure and hence the conformational rigidity, which can increase the resistance to enzymatic degradation [15–19]. Such units are also found in biologically interesting natural products, such as the antibacterial fumimycin [20].

Herein, we describe our endeavours towards the synthesis of such valuable compounds by utilizing the multicomponent aryne reaction.

## Results and Discussion

### Formation of quaternary Schöllkopf adducts

**Scheme 5:**
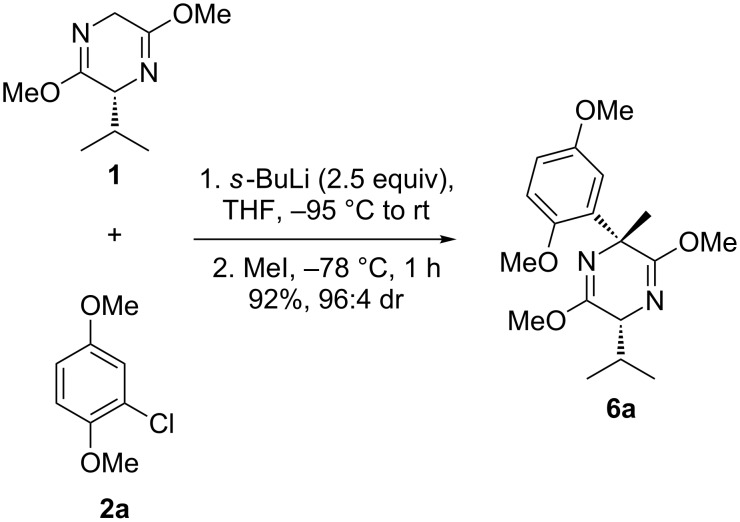
Formation of α-methyl, α-aryl Schöllkopf adduct.

The same procedure was carried out as for the α-arylation reactions: The benzyne precursor **2a** and Schöllkopf’s bislactim ether **1** were allowed to react with 2.5 equivalents of *sec*-butyllithium at –95 °C to carry out the required *ortho*-lithiation for benzyne formation from **2a** and the deprotonation of bislactim **1**. During warming to room temperature, fragmentation to dimethoxybenzyne occurred and nucleophilic attack of the Schöllkopf auxiliary took place. Addition of four equivalents of iodomethane at this temperature and stirring for 1 h gave the desired quaternary species **6a** in 88% yield with a dr of 85:15. Lowering the quench temperature to –78 °C increased both the yield and the dr to 92% and 96:4, respectively (Scheme 5).

**Figure 1:**
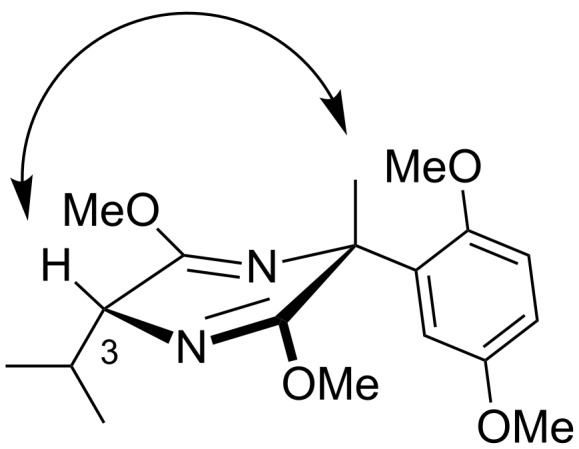
NOESY correlation observed for **6a**.

The major diastereoisomer was confirmed to have the isopropyl and aryl moieties *syn*, by ^1^H NMR NOESY analysis; a clear NOESY correlation was observed between the methyl group and the proton at C-3 (Figure 1).

The scope of the reaction was then examined to determine which electrophiles could be introduced at C-6 (Table 1).

**Table 1:** Formation of quaternary Schöllkopf adducts employing a range of electrophiles.

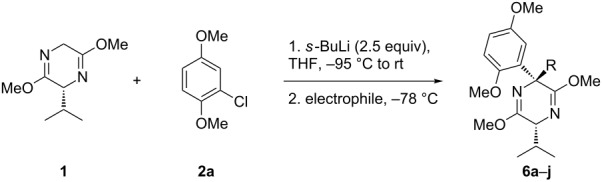						

Entry^a^	Electrophile	R	Time (h)^b^	Product	Yield (%)^c^	dr^d^

1	MeI	Me	1	**6a**	92	96:4
2	BnBr	Bn	6	**6b**	88	>98:2
3	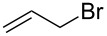	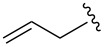	1	**6c**	85	>98:2
4	MOMCl	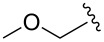	1	**6d**	53	95:5
5	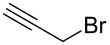	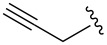	1	**6e**	80	>98:2
6	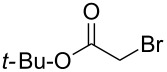	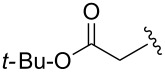	3	**6f**	51	>98:2
7	AcCl	COMe	6	**6g**	59	89:11
8	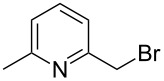	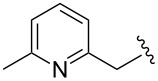	5	**6h**	71	>98:2
9	PhCHO	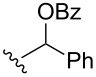	12	**6i**^e^	69	>98:2^f^
10	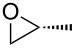 BF_3_·OEt_2_	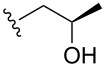	1	**6j**	50	>98:2

^a^Reactions were carried out on a 1 mmol scale in THF (0.2 M) with 1.5 equiv of halide **2a** and 2.75 equiv *s*-BuLi at –95 °C, followed by warming to room temperature over 18 h, recooling to –78 °C, and the addition of 4 equiv of electrophile. ^b^Reactions were monitored by GC–MS. ^c^Isolated yield. ^d^Determined by ^1^H NMR integration. ^e^**6i** was isolated as its benzoyl ester, due to retro-aldol reactions occurring when the free alcohol was subjected to flash-column chromatography. ^f^The adduct **6i** was obtained as a mixture of epimeric benzylic alcohols (1:1).

**Figure 2:**
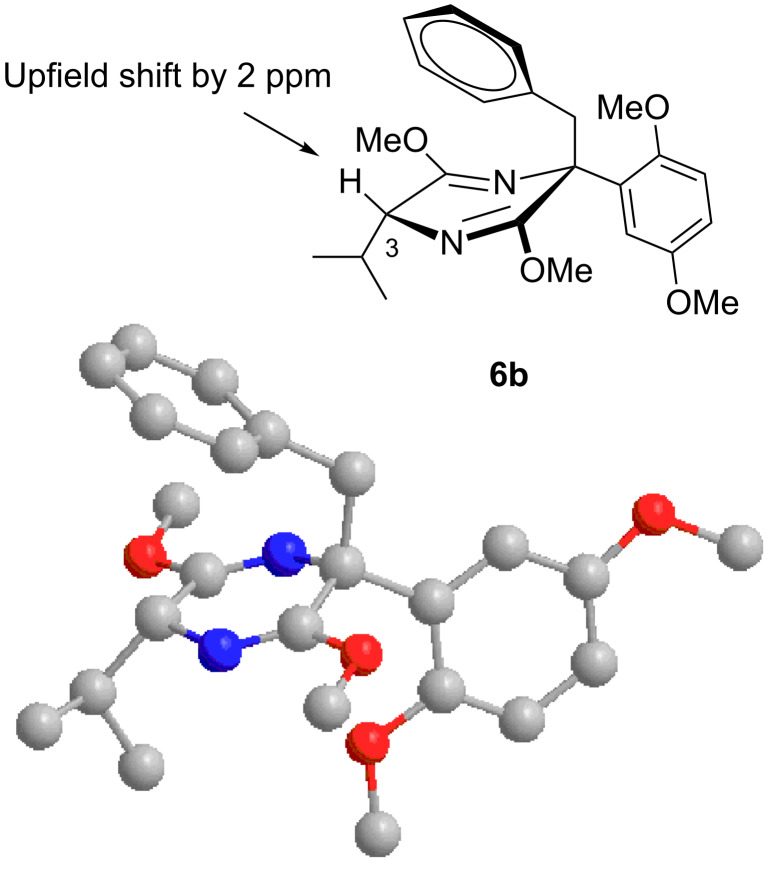
X-ray crystal structure of **6b**.

Alkylations employing benzyl bromide, allyl bromide and propargyl bromide (Table 1, entries 2, 3 and 5) proceeded smoothly, furnishing the desired products **6b**, **6c** and **6e** in excellent yields and high diastereoselectivity. Interestingly, adduct **6b** displays a large upfield shift (around 2 ppm) at the C-3 proton compared to other analogues. This indicates that the product has adopted an “aryl-inside” conformation in which the C-3 proton is situated in the shielding cone of the aromatic ring [21]. A single-crystal X-ray crystallographic analysis confirmed the presence of this conformation, at least in the crystal lattice (Figure 2).

**Figure 3:**
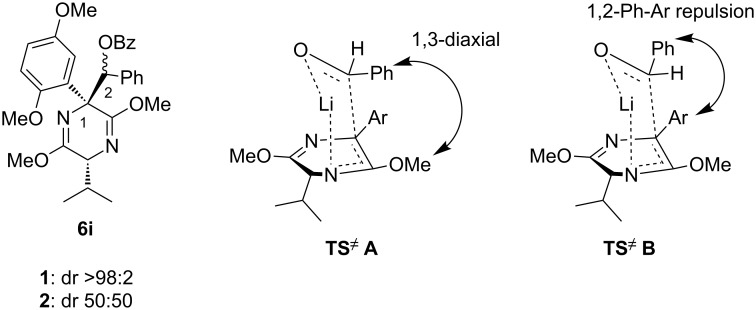
Transition state analysis to explain the lack of diastereoselectivity at C-2.

Further alkylations with methyl chloromethyl ether, *tert*-butyl bromoacetate and 2-(bromomethyl)-6-methylpyridine (Table 1, entries 4, 6 and 8) provided adducts **6d**, **6f** and **6h** respectively, also with high diastereoselectivity, but in more modest yields. Acylation with acetyl chloride (Table 1, entry 7) afforded ketone **6g** in 59% yield, with a lower dr of 89:11, most likely due to the less sterically hindered nature of this electrophile. Replacing acetyl chloride with its corresponding Weinreb amide gave none of the required product. An aldol type reaction with benzaldehyde (Table 1, entry 9) afforded alcohol **6i** in good yield, with complete diastereoselectivity at C-6, but no selectivity at the alcohol stereocenter (1:1). Examination of the two possible Zimmerman–Traxler type transition states for such reactions (Figure 3), as proposed by Schöllkopf [22], reveals that **TS^≠ ^A**, with the phenyl group axial, is consistent with there being an unfavourable 1,3-diaxial interaction with the methoxy group. However, there is an equally unfavourable interaction in **TS^≠^ B** where the phenyl group occupies the equatorial position, as there is now a 1,2-gauche interaction with the bulky di-methoxyaryl group adjacent to it. We consider that neither of these transition states is lower in energy, which would be consistent with the lack of diastereoselectivity.

Finally, the addition of (*R*)-propylene oxide as the electrophile in the presence of boron trifluoride etherate (Table 1, entry 10) gave alcohol **6j**. When (*S*)-propylene oxide was used instead there was no reaction, and indeed Schöllkopf noted significant levels of kinetic resolution when such reactions were performed with racemic oxiranes [23].

**Scheme 6:**
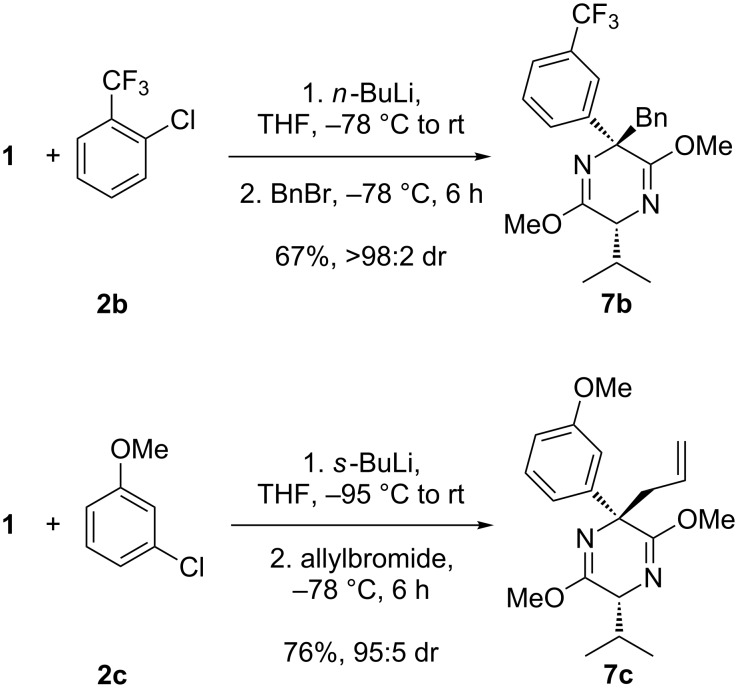
Formation of quaternary adducts.

In our previous publication, we demonstrated the scope of the methodology over a range of *ortho*-lithiation benzyne precursors. To establish that any one of these precursors could be used to form quaternary adducts, we subjected the benzylation conditions to precursor **2b** and the allylation conditions to precursor **2c**, affording adducts **7b** and **7c**, respectively, again in good yields and dr’s and with complete regioselectivity for these asymmetric benzynes (Scheme 6).

### Hydrolysis of quaternary adducts

**Scheme 7:**
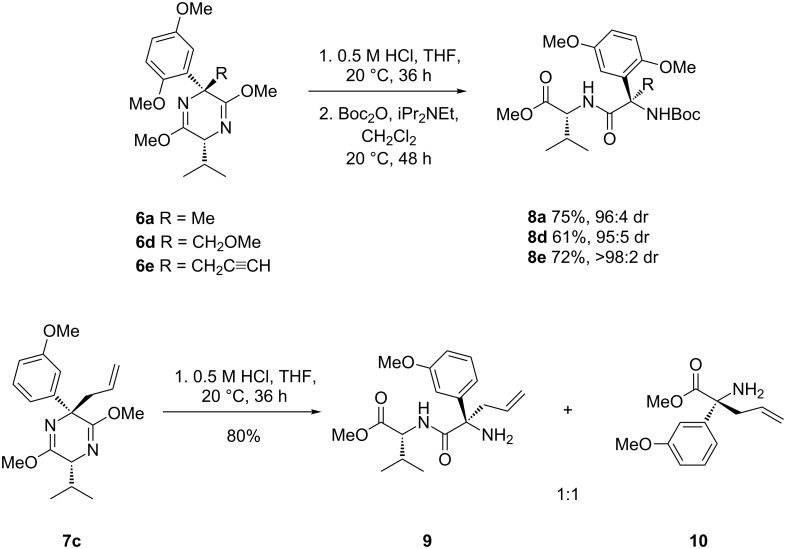
Hydrolysis of quaternary adducts.

The next task to be undertaken was the hydrolysis of the sterically restricted quaternary bislactim ethers. A selection of the relatively less bulky products, **6a**, **6d** and **6e**, was subjected to standard, mild hydrolysis conditions, employing 0.5 M HCl in THF at room temperature [12], affording the desired quaternary methyl esters as their corresponding dipeptides, **8a**, **8d** and **8e**, in good yields (Scheme 7). The less shielded imidate group underwent complete hydrolysis, but the imidate functional group α to the quaternary center experienced incomplete cleavage to the peptide bond, a trend which is common in the literature for quaternary adducts [21,24–26]. Subjecting analogue **7c**, with just one methoxy group on the aromatic ring, to the same conditions yielded the dipeptide **9** and the desired methyl ester **10** in a 1:1 ratio (Scheme 7).

**Scheme 8:**
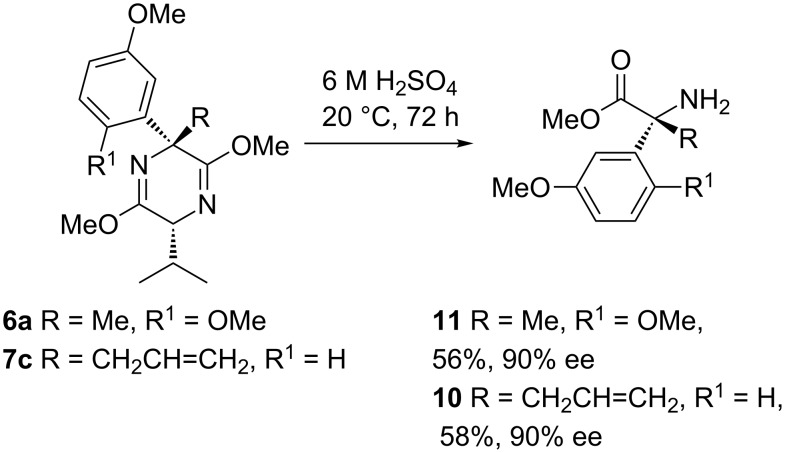
Hydrolysis to amino acids.

Screening of various acidic hydrolysis reaction conditions in these systems led to the observation that when either **6a** or **7c** was stirred in neat 6 M sulfuric acid for three days, the required constituent amino acid **11** or **10** was isolated in reasonable yields (Scheme 8). Chiral HPLC analysis confirmed the expected high enantioselectivity of these esters.

These results are promising, as they demonstrate that if the aryl group is derived from a less sterically hindered benzyne precursor, hydrolysis is more facile, as was observed by Lee [27] for unsubstituted aryl, quaternary bislactim ethers. Using high concentrations of acids with more-nucleophilic conjugate bases resulted in the predominate formation of diketopiperazines, and low concentrations of all of the acids tested gave the dipeptide as the major product for all analogues with two methoxy groups (**6a**–**6i**).

**Scheme 9:**
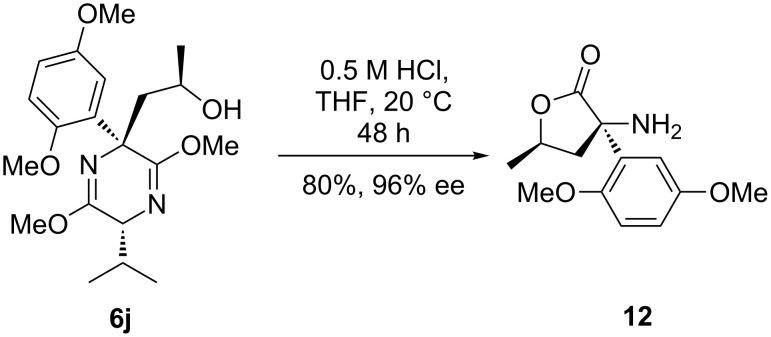
Hydrolysis of analogue **6j**.

The only example where the use of low concentrations of acid coupled with a dimethoxy aryl group resulted in the principal formation of the desired ester was in the case of alcohol **6j**. Mild acidic hydrolysis with 0.5 M HCl gave lactone **12** in 80% yield and 96% ee (Scheme 9). The ability of the alcohol to undergo lactonization under the reaction conditions clearly aided the hydrolysis of this more sterically hindered imidate.

**Scheme 10:**
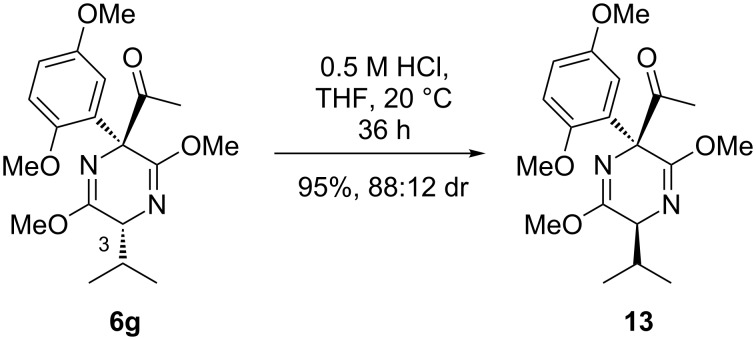
Epimerization at C-3 of **6g**.

We assumed that ketone **6g** would also undergo hydrolysis due to its nonbulky nature. However, upon exposure to 0.5 M HCl, epimerization of the isopropyl group at C-3 occurs in near quantitative yield, giving adduct **13** (Scheme 10). The electron-withdrawing effect of the carbonyl moiety must increase the acidity of the C-3 proton to such an extent that the acidic media simply epimerizes at this center to afford the more thermodynamically stable *anti*-adduct.

## Conclusion

The extension of the aryne α-arylation methodology allowed for the synthesis of quaternary aryl bislactim ethers in good to excellent yields and high diastereoselectivity by means of a three-component coupling reaction in which the reaction of an electrophile with the intermediate benzylic anion gave a range of C–alkyl and hydroxyalkyl derivatives. Hydrolysis of these quaternary adducts gave the constituent amino acid methyl esters in high enantioselectivity for less substituted compounds. Adducts with two methyl ethers on the aryl unit underwent hydrolysis to the corresponding valine dipeptides.

## Supporting Information

File 1 Detailed experimental procedures and analytical data for compounds **6a**–**j**, **7b**–**c**, **8a**, **8d**, **8e** and **9**–**13**.

File 2 NMR spectral data for compounds **6a**–**j** and **7b**–**c**.
