# Supplementary material for: Asymmetric synthesis of quaternary aryl amino acid derivatives via a three-component aryne coupling reaction
Source: Beilstein J Org Chem. 2011 Nov 25;7:1570–6. doi: 10.3762/bjoc.7.185 (PMC3252860; doi:10.3762/bjoc.7.185)
Supplement: File 1 — Detailed experimental procedures and analytical data for compounds 6a–j, 7b–c, 8a, 8d, 8e and 9–13. [file Beilstein_J_Org_Chem-07-1570-s001.pdf]

Supporting Information File 1

for

Asymmetric synthesis of quaternary aryl amino acid derivatives in a three-component aryne coupling reaction

Elizabeth P. Jones[1], Peter Jones[2], Andrew J. P. White[1] and Anthony G. M. Barrett*[1]

Address: [1]Department of Chemistry, Imperial College London, London, SW7 2AZ, England and [2]Worldwide Medicinal Chemistry, Pfizer Limited, Ramsgate Road, Sandwich, Kent, CT13 9NJ, England

Email: Anthony G. M. Barrett*[1] - agm.barrett@imperial.ac.uk

* Corresponding author

Detailed experimental procedures and analytical data for compounds 6a–j, 7b–c, 8a, 8d, 8e and 9–13

Table of contents

1. General methods..2
2. General procedure for the synthesis of quaternary Schöllkopf adducts 2
3. Hydrolysis products...9

1. General methods

All mixtures were carried out in oven-dried glassware under N_2 with solvents and reagents as commercially supplied, unless otherwise stated. THF and CH_2Cl_2 were redistilled from Na-Ph_2CO and CaH_2, respectively. Thin layer chromatography was performed on precoated silica gel F_{254} glass plates with visualization under UV light or by staining with potassium permanganate solution, ninhydrin or Dragendorff's reagent. Flash column chromatography was either performed over silica gel, particle size 40–63 μm, or on prepacked Redisep cartridges (eluants are given in parenthesis). Melting points were determined by using a hot-stage apparatus and are uncorrected. IR spectra were recorded as thin films and the absorption bands are reported in wavenumbers (cm^{-1}). ^{1}H NMR spectra were recorded at 400 and 500 MHz and referenced to the residual solvent peak at 7.26 ppm ($CDCl_3$) and are quoted in ppm to two decimal places with coupling constants (J) to the nearest 0.1 Hz. ^{13}C NMR were recorded at 100 and 125 MHz and referenced to the solvent at 77.0 ppm ($CDCl_3$) and are quoted in ppm to one decimal place.

2. General procedure for the synthesis of quaternary Schöllkopf adducts

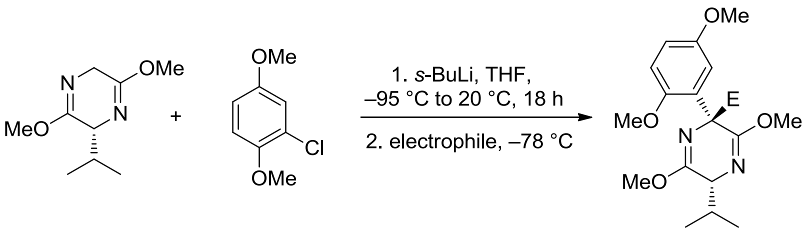

1 **2a** **6a–j**

General procedure: *sec*-Butyllithium (in cyclohexane 1.28 M; 2.15 mL, 2.75 mmol) was added dropwise with stirring to imidate **1** (0.18 mL, 1.00 mmol) and 2-chloro-1,4-dimethoxybenzene (**2a**) (0.21 mL, 1.50 mmol) in THF (5 mL) at −95 °C. The mixture was maintained at −95 °C for 30 min, before being allowed to warm to room temperature overnight. The mixture was recooled to −78 °C when the electrophile (4 equiv) was added and stirring was continued at −78 °C until GCMS analysis indicated that the reaction was complete. The mixture was warmed to room temperature and H_2O (5 mL) was added and the mixture extracted with EtOAc (15 mL × 3). The combined organic layers were washed with brine (5 mL), dried ($MgSO_4$), filtered and rotary evaporated.

(2*R*,5*R*)-2-(2,5-Dimethoxyphenyl)-5-isopropyl-3,6-dimethoxy-2-methyl-2,5-dihydropyrazine (6a)

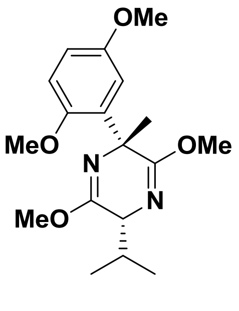

6a

Imidate **6a** was prepared according to the general procedure, employing MeI (0.25 mL, 4.00 mmol) as the electrophile at −78 °C for 1 h. Chromatography (EtOAc:hexanes 1:15 to 1:8) gave **6a** (0.307 g, 94:6 dr, 92%) as an off-white solid: mp 32–34 °C (EtOAc/hexanes); R_f 0.55 (EtOAc:hexanes 1:4); $[\alpha]_D^{25}$ = −88.3 (*c* 1.2, $CHCl_3$); IR 1688 (C=N), 1671 (C=N), 1497, 1462, 1224, 1201 cm^{-1}; Major Diastereoisomer: ^{1}H NMR (400 MHz, $CDCl_3$) δ 7.03 (d, 1H, *J* = 2.3 Hz, Ar-H), 6.80 (m, 2H, Ar-H), 3.95 (d, 1H, *J* = 3.2 Hz, C<u>H</u>-iPr), 3.81 (s, 3H, OMe), 3.64 (s, 6H, 2 × OMe), 3.64 (s, 3H, OMe), 2.47 (dsept, 1H, *J* = 6.8, 3.2 Hz, iPr), 1.64 (s, 3H, CH_3), 1.20 (d, 3H, *J* = 6.9 Hz, iPr), 0.87 (d, 3H, *J* = 6.7 Hz, iPr); ^{13}C NMR (125 MHz, $CDCl_3$) δ 166.5, 162.4, 153.2, 152.3, 133.9, 113.9, 112.6, 111.8, 59.9, 58.8, 56.0, 55.5, 52.7, 52.4, 30.0, 26.1, 19.8, 17.1; HRMS (ESI) calcd. $C_{18}H_{27}N_2O_4$: $(M + H)^+$, 335.1971; found: $(M + H)^+$, 335.1979.

(2*R*,5*R*)-2-Benzyl-2-(2,5-dimethoxyphenyl)-5-isopropyl-3,6-dimethoxy-2,5-dihydropyrazine (6b)

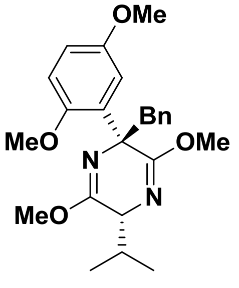

6b

Imidate **6b** was prepared according to the general procedure, employing $PhCH_2Br$ (0.48 mL, 4.00 mmol) as the electrophile at −78 °C for 6 h. Chromatography (EtOAc:hexanes 1:15 to 1:8) gave **6b** (0.360 g, >98:2 dr, 88%) as a yellow solid: mp 86–88 °C (EtOAc/hexanes); R_f 0.4 (EtOAc:hexanes 1:3); $[\alpha]_D^{25}$ = 9.6 (*c* 0.8, $CHCl_3$); IR 1692 (C=N), 1668 (C=N), 1497, 1461, 1299, 1230, 1193 cm^{-1}; ^{1}H NMR (400 MHz, $CDCl_3$) δ 7.24 (dd, 3H, *J* = 5.1, 2.1 Hz, Ar-H), 7.19 (d, 1H, *J* = 2.7 Hz, Ar-H), 7.07 (m, 2H, Ar-H), 6.85 (m, 2H, Ar-H), 3.84 (s, 3H, OMe), 3.70 (s, 3H, OMe), 3.67 (s, 3H, OMe), 3.66 (s, 3H, OMe), 3.48 (AB, 2H, *J* = 12.2 Hz, CH_2Ph), 2.11 (d, 1H, *J* = 3.0 Hz, C<u>H</u>-iPr), 2.08 (m, 1H, iPr), 0.89 (d, 3H, *J* = 6.7 Hz, iPr), 0.71 (d, 3H, *J* = 6.6 Hz, iPr); ^{13}C NMR (125 MHz, $CDCl_3$) δ 163.7, 162.3, 153.2, 152.5, 136.3, 134.0, 131.3, 127.5, 126.5, 114.2, 113.1, 112.0, 63.3, 60.1, 56.1, 55.6, 52.6, 52.2, 44.3, 29.8, 19.5, 17.4; HRMS (ESI) calcd. $C_{24}H_{31}N_2O_4$: $(M + H)^+$, 411.2284; found: $(M + H)^+$, 411.2272.

(2*R*,5*R*)-2-Allyl-2-(2,5-dimethoxyphenyl)-5-isopropyl-3,6-dimethoxy-2,5-dihydropyrazine (6c)

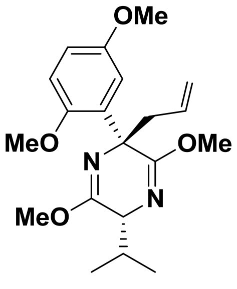

6c

Imidate **6c** was prepared according to the general procedure, by employing allyl bromide (0.35 mL, 4.00 mmol) as the electrophile at −78 °C for 1 h. Chromatography (EtOAc:hexanes 1:15 to 1:10) gave **6c** (0.305 g, >98:2 dr, 85%) as a colourless oil; R_f 0.52 (EtOAc:hexanes 1:3); $[\alpha]_D^{25}$ = −59.5 (*c* 1.0, $CHCl_3$); IR 1689 (C=N), 1670 (C=N), 1498, 1299, 1227, 1140 cm^{-1}; ^1H NMR (400 MHz, $CDCl_3$) δ 7.04 (d, 1H, *J* = 2.7 Hz, Ar-H), 6.81 (m, 2H, Ar-H), 5.73 (m, 1H, $C\underline{H}=CH_2$), 5.11 (m, 2H, $CH=C\underline{H_2}$), 3.88 (d, 1H, *J* = 3.5 Hz, $C\underline{H}$-iPr), 3.80 (s, 3H, OMe), 3.68 (s, 3H, OMe), 3.67 (s, 3H, OMe), 3.66 (s, 3H, OMe), 2.96 (dd, 1H, *J* = 13.1, 7.1 Hz, $C\underline{H}_2CH=CH_2$), 2.91 (dd, 1H, *J* = 13.0, 7.7 Hz, $C\underline{H}_2CH=CH_2$), 2.40 (dsept, 1H, *J* = 6.9, 3.7 Hz, iPr), 1.17 (d, 3H, *J* = 6.9 Hz, iPr), 0.86 (d, 3H, *J* = 6.8 Hz, iPr); ^{13}C NMR (125 MHz, $CDCl_3$) δ 163.9, 163.2, 153.2, 152.4, 133.4, 133.3, 119.1, 114.4, 113.1, 112.2, 62.2, 61.2, 56.1, 55.6, 52.7, 52.4, 43.8, 30.5, 19.9, 17.7; HRMS (ESI) calcd. $C_{20}H_{29}N_2O_4$: $(M + H)^+$, 361.2127; found: $(M + H)^+$, 361.2112.

(2*S*,5*R*)-2-(2,5-Dimethoxyphenyl)-5-isopropyl-3,6-dimethoxy-2-(methoxymethyl)-2,5-dihydropyrazine (6d)

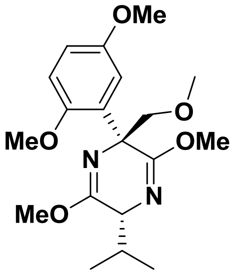

6d

Imidate **6d** was prepared according to the general procedure, by employing MOMCl (0.30 mL, 4.00 mmol) as the electrophile at −78 °C for 1 h. Chromatography (EtOAc:hexanes 1:15 to 1:5) gave **6d** (0.20 g, 95:5 dr, 53%) as an off-white solid: mp 48–50 °C (EtOAc/hexanes); R_f 0.29 (EtOAc:hexanes 1:4); $[\alpha]_D^{25}$ = −64.2 (*c* 1.1, $CHCl_3$); IR 1696 (C=N), 1501, 1464, 1230, 1136 cm^{-1}; Major diastereoisomer: ^1H NMR (400 MHz, $CDCl_3$) δ 6.81 (m, 3H, Ar-H), 4.11 (d, 1H, *J* = 8.9 Hz, $C\underline{H}_2OMe$), 4.02 (d, 1H, *J* = 3.3 Hz, $C\underline{H}$-iPr), 3.86 (d, 1H, *J* = 8.9 Hz, $C\underline{H}_2OMe$), 3.78 (s, 3H, OMe), 3.67 (s, 9H, 3 × OMe), 3.38 (s, 3H, $CH_2OC\underline{H_3}$), 2.43 (dsept, 1H, *J* = 6.8, 3.3 Hz, iPr), 1.18 (d, 3H, *J* = 6.9 Hz, iPr), 0.84 (d, 3H, *J* = 6.7 Hz, iPr); ^{13}C NMR (125 MHz, $CDCl_3$) δ 164.4, 162.9, 153.2, 152.3, 131.5, 113.9, 113.2, 112.2, 77.8, 63.0, 61.0, 59.8, 56.3, 55.6, 52.7, 52.6, 30.3, 19.8, 17.4; HRMS (ESI) calcd. $C_{19}H_{29}N_2O_5$: $(M + H)^+$, 365.2076; found: $(M + H)^+$, 365.2071.

(2*R*,5*R*)-2-(2,5-Dimethoxyphenyl)-5-isopropyl-3,6-dimethoxy-2-(prop-2-ynyl)-2,5-dihydropyrazine (6e)

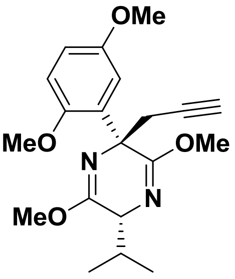

6e

Imidate **6e** was prepared according to the general procedure, by employing propargyl bromide (0.59 g of an 80% wt in toluene solution, 4.00 mmol) as the electrophile at −78 °C for 1 h. Chromatography (EtOAc:hexanes 1:10 to 1:5) gave **6e** (0.285 g, >98:2 dr, 80%) as a white solid: mp 59–60 °C(EtOAc/hexanes); R_f 0.38 (EtOAc:hexanes 1:4); $[\alpha]_D^{25}$ = −58.8 (*c* 1.3, $CHCl_3$); IR 1692 (C=N), 1667 (C=N), 1500, 1228, 1033 cm^{-1}; ^1H NMR (400 MHz, $CDCl_3$) δ 6.84 (m, 3H, Ar-H), 4.16 (d, 1H, *J* = 3.6 Hz, CH-iPr), 3.78 (s, 3H, OMe), 3.72 (s, 6H, 2 × OMe), 3.71 (s, 3H, OMe), 3.21 (dd, 1H, *J* = 16.1, 2.6 Hz, $CH_2C≡CH$), 3.16 (dd, 1H, *J* = 16.1, 2.6 Hz, $CH_2C≡C$), 2.39 (dsept, 1H, *J* = 6.8, 3.6 Hz, iPr), 1.97 (t, 1H, *J* = 2.6 Hz, C≡CH), 1.18 (d, 3H, *J* = 6.9 Hz, iPr), 0.84 (d, 3H, *J* = 6.8 Hz, iPr); ^{13}C NMR (125 MHz, $CDCl_3$) δ 164.2, 163.0, 153.2, 152.3, 132.1, 144.5, 113.8, 112.6, 80.7, 70.7, 62.5, 61.3, 56.5, 55.6, 52.8, 52.7, 30.7, 19.9, 17.7; HRMS (ESI) calcd. $C_{20}H_{27}N_2O_4$: $(M + H)^+$, 359.1971; found: $(M + H)^+$, 359.1967.

***tert*-Butyl 2-((2*R*,5*R*)-2-(2,5-dimethoxyphenyl)-5-isopropyl-3,6-dimethoxy-2,5-dihydropyrazin-2-yl)acetate (6f)**

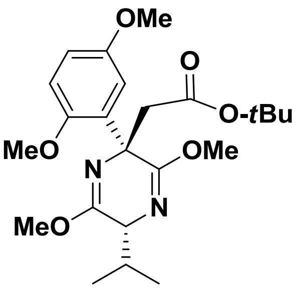

6f

Imidate **6f** was prepared according to the general procedure, by employing *tert*-butyl bromoacetate (0.59 mL, 4.00 mmol) as the electrophile at −78 °C for 3 h. Chromatography (EtOAc:hexanes 1:10 to 1:5) gave **6f** (0.220 g, >98:2 dr, 51%) as a colourless oil; R_f 0.52 (EtOAc:hexanes 1:4); $[\alpha]_D^{25}$ = −43.3 (*c* 0.9, $CHCl_3$); IR 1728 (C=O),1691 (C=N),1498, 1356, 1230, 1153 cm^{-1}; ^1H NMR (400 MHz, $CDCl_3$) δ 6.86 (m, 1H, Ar-H), 6.77 (m, 2H, Ar-H), 3.96 (d, 1H, *J* = 4.6 Hz, CH-iPr), 3.78 (s, 3H, OMe), 3.75 (s, 3H, OMe), 3.72 (s, 6H, 2 × OMe), 3.32 (AB quartet, 2H, *J* = 14.1 Hz, CH_2Ph), 2.20 (m, 1H, iPr), 1.40 (s, 9H, *t*-Bu),1.08 (d, 3H, *J* = 6.8 Hz, iPr), 0.80 (d, 3H, *J* = 6.8 Hz, iPr); ^{13}C NMR (125 MHz, $CDCl_3$) δ 170.2, 164.1, 162.6, 153.2, 152.4, 132.9, 114.8, 114.7, 113.0, 79.9, 62.2, 61.4, 57.0, 55.6, 52.6, 52.5, 45.1, 31.3, 27.9, 19.9, 18.4; HRMS (ESI) calcd. $C_{23}H_{35}N_2O_6$: $(M + H)^+$, 435.2495; found: $(M + H)^+$, 435.2485.

1-((2*R*,5*R*)-2-(2,5-Dimethoxyphenyl)-5-isopropyl-3,6-dimethoxy-2,5-dihydropyrazin-2-yl)ethanone (6g)

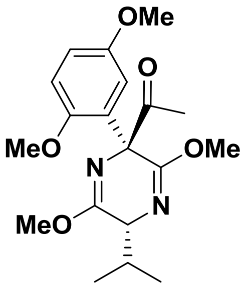

6g

Imidate **6g** was prepared according to the general procedure, by employing acetyl chloride (0.29 mL, 4.00 mmol) as the electrophile at −78 °C for 3 h. Chromatography (EtOAc:hexanes 1:5) gave **6g** (0.215 g, 89:11 dr, 59%) as a yellow solid: mp 83–84 °C (EtOAc/hexanes); R_f 0.33 (EtOAc:hexanes 1:4); $[\alpha]_D^{25}$ = −183.3 (*c* 0.9, $CHCl_3$); IR 1679 (C=N), 1630 (C=O), 1487, 1356, 1209, 1176 cm^{-1}; ^1H NMR (400 MHz, $CDCl_3$) δ 7.05 (d, 1H, *J* = 2.9 Hz, Ar-H), 6.87 (m, 2H, Ar-H), 4.80 (d, 1H, *J* = 10.3 Hz, C$\underline{H}$-iPr), 3.82 (s, 3H, OMe), 3.80 (s, 3H, OMe), 3.79 (s, 3H, OMe), 3.51 (s, 3H, OMe), 2.28 (s, 3H, C$\underline{H_3}$), 2.39 (dsept, 1H, *J* = 10.3, 6.7 Hz, iPr), 1.01 (d, 6H, *J* = 6.7 Hz, iPr); ^{13}C NMR (125 MHz, $CDCl_3$) δ 172.1, 160.5, 153.5, 151.5, 136.0, 126.3, 116.9, 115.7, 113.7, 112.5, 58.3, 57.2, 56.4, 55.8, 53.6, 26.9, 23.3, 19.3, 18.8; HRMS (ESI) calcd. $C_{19}H_{27}N_2O_5$: $(M + H)^+$, 363.1920; found: $(M + H)^+$, 363.1912.

(2*R*,5*R*)-2-(2,5-Dimethoxyphenyl)-5-isopropyl-3,6-dimethoxy-2-((6-methylpyridin-2-yl)methyl)-2,5-dihydropyrazine (6h)

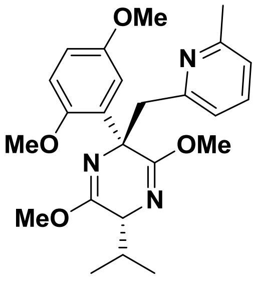

6h

Imidate **6h** was prepared according to the general procedure, by employing 2-(bromomethyl)-6-methylpyridine (0.74 g, 4.00 mmol) in THF (5 mL) as the electrophile at −78 °C for 5 h. Chromatography (EtOAc:hexanes 1:5 to 1:1) gave **6h** (0.300 g, >98:2 dr, 71%) as a yellow oil; R_f 0.17 (EtOAc:hexanes 1:4); $[\alpha]_D^{25}$ = 3.4 (*c* 1.0, $CHCl_3$); IR 1692 (C=N),1668 (C=N),1497, 1356, 1227, 1031 cm^{-1}; ^1H NMR (400 MHz, $CDCl_3$) δ 7.43 (t, 1H, *J* = 7.6 Hz, Ar-H), 7.18 (d, 1H, *J* = 2.7 Hz, Ar-H), 7.01 (d, 1H, *J* = 7.6, Ar-H), 6.84 (m, 3H, Ar-H), 3.82 (s, 3H, OMe), 3.70 (s, 3H, OMe), 3.67 (s, 3H, OMe), 3.66 (s, 3H, OMe), 3.64 (AB quartet, 2H, *J* = 12.0 Hz, CH_2Ph), 2.51 (s, 3H, CH_3), 2.17 (d, 1H, *J* = 3.2 Hz, C$\underline{H}$-iPr), 2.13 (m, 1H, iPr), 0.93 (d, 3H, *J* = 6.8 Hz, iPr), 0.73 (d, 3H, *J* = 6.7 Hz, iPr); ^{13}C NMR (125 MHz, $CDCl_3$) δ 163.3, 162.5, 157.4, 156.6, 153.3, 152.5, 135.5, 133.8, 122.4, 120.6, 114.1, 113.3, 112.5, 63.2, 60.0, 56.2, 55.6, 52.6, 52.4, 46.5, 29.8, 24.4, 19.6, 17.4; HRMS (ESI) calcd. $C_{24}H_{32}N_3O_4$: $(M + H)^+$, 426.2393; found: $(M + H)^+$, 426.2372.

((2*S*,5*R*)-2-(2,5-Dimethoxyphenyl)-5-isopropyl-3,6-dimethoxy-2,5-dihydropyrazin-2-yl)(phenyl)methyl benzoate (6i)

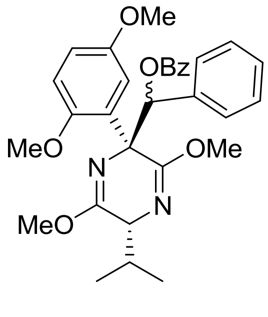

6i

Imidate **6i** was prepared according to the general procedure, by employing PhCHO (0.41 mL, 4.00 mmol) as the electrophile at −78 °C for 12 h. The residue was then taken up in CH_2Cl_2 (5 mL) and benzoyl chloride (0.23 mL, 2 mmol) and dimethylamin-pyridine (0.61 g, 5 mmol) was added, before the mixture was stirred at room temperature overnight. The mixture was quenched with H_2O (5 mL) and extracted with EtOAc (2 × 5 mL), before being dried ($MgSO_4$), filtered and rotary evaporated. Chromatography (PhMe) gave **6i** (0.366 g, >98:2 dr, 69%) as a colourless oil and a 1:1 mixture of diastereoisomers at OBz; R_f 0.51(PhMe); $[\alpha]_D^{25}$ = −103.8 (*c* 1.0, $CHCl_3$); IR 1722 (C=O), 1691(C=N), 1498, 1273, 1238, 1229 cm^{-1}; ^1H NMR (400 MHz, $CDCl_3$) δ 8.02 (d, 2H, *J* = 7.3 Hz, Ar-H), 7.94 (d, 2H, *J* = 7.4 Hz, Ar-H), 7.51 (m, 2H, Ar-H), 7.38 (m, 4H, Ar-H), 7.36 (s, 1H, C<u>H</u>OBz), 7.33 (s, 1H, C<u>H</u>OBz), 7.27 (m, 6H, Ar-H), 7.19 (m, 4H, Ar-H), 6.95 (d, 1H, *J* = 3.0 Hz, Ar-H), 6.92 (d, 1H, *J* = 2.5 Hz, Ar-H), 6.80 (m, 4H, Ar-H), 3.91 (s, 3H, OMe), 3.88 (s, 3H, OMe), 3.81 (d, 1H, *J* = 3.8 Hz, CHiPr), 3.71 (s, 3H, OMe), 3.70 (s, 6H, 2 × OMe), 3.68 (s, 3H, OMe), 3.67 (s, 3H, OMe), 3.66 (s, 3H, OMe), 2.58 (d, 1H, *J* = 3.4 Hz, CH*iPr*), 2.17 (m, 1H, iPr), 2.06 (m, 1H, iPr), 0.94 (d, 3H, *J* = 6.9 Hz, iPr), 0.91(d, 3H, *J* = 6.8 Hz, iPr), 0.71(d, 3H, *J* = 6.7 Hz, iPr), −0.14 (d, 3H, *J* = 6.8 Hz, iPr); ^{13}C NMR (125 MHz, $CDCl_3$) δ 166.0, 165.9, 164.4, 164.3, 160.9, 159.6, 153.3, 153.0, 152.6, 152.6, 137.8, 137.7, 137.4, 132.7, 132.7, 130.8, 130.7, 129.8, 128.7, 129.1, 128.6, 128.3, 128.0, 127.8, 127.8, 127.4, 125.3, 116.2, 114.5, 114.0, 113.7, 113.4, 113.3, 78.4, 77.1, 68.1, 66.7, 60.4, 59.6, 56.9, 56.4, 55.6, 55.5, 52.8, 52.6, 52.4, 52.3, 30.4, 29.8, 21.5, 19.5, 17.4, 15.5. HRMS (ESI) calcd. $C_{31}H_{35}N_2O_6$: $(M + H)^+$, 531.2495; found: $(M + H)^+$, 531.2485.

(*R*)-1-((2*R*,5*R*)-2-(2,5-Dimethoxyphenyl)-5-isopropyl-3,6-dimethoxy-2,5-dihydropyrazin-2-yl)propan-2-ol (6j)

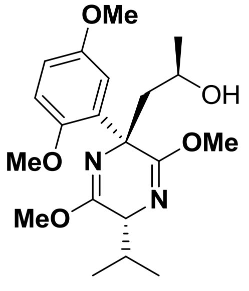

6j

Imidate **6j** was prepared according to the general procedure, by employing (*R*)-propylene oxide (0.28 mL, 4.00 mmol) and $BF_3 \cdot OEt_2$ (0.49 mL, 4.00 mmol) as the electrophile at −78 °C for 1 h. Chromatography (EtOAc:hexanes 1:5 to 1:1) gave **6j** (0.186 g, >98:2 dr, 50%) as a yellow oil; R_f 0.21 (EtOAc:hexanes 1:3);

[α]$_D^{25}$ = −61 °C (*c* 0.7, $CHCl_3$); IR 3433 (OH), 1689 (C=N), 1670 (C=N),1498, 1229, 1050 cm^{-1}; ^{1}H NMR (400 MHz, $CDCl_3$) δ 6.84 (m, 2H, Ar-H), 6.77 (dd, 1H, *J* = 8.9, 3.0 Hz, Ar-H), 4.07 (br s, 1H, OH), 7.23 (d, 1H, *J* = 3.9 Hz, CHiPr), 3.80 (m, 1H, C<u>H</u>OH), 3.80 (s, 3H, OMe), 3.77 (s, 3H, OMe), 3.77 (s, 3H, OMe), 3.76 (s, 3H, OMe), 2.41 (m, 2H, CH_2), 1.62 (m, 1H, iPr); ^{13}C NMR (125 MHz, $CDCl_3$) δ 167.5, 165.0, 153.2, 151.2, 134.5, 114.6, 113.9, 112.8, 66.1, 64.4, 63.1, 56.6, 55.7, 53.0, 52.8, 47.5, 31.5, 23.6, 20.1, 19.4; HRMS (ESI) calcd. $C_{20}H_{31}N_2O_5$: $(M + H)^+$, 379.2233; found: $(M + H)^+$, 379.2217.

(2*R*,5*R*)-2-Benzyl-5-isopropyl-3,6-dimethoxy-2-(3-(trifluoromethyl)phenyl)-2,5-dihydropyrazine (7b)

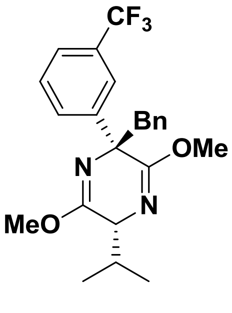

7b

n-Butyllithium (in hexanes 1.4 M; 2.14 mL, 3.00 mmol) was added dropwise to imidate **1** (0.18 mL, 1.00 mmol) and 1-chloro-2-(trifluoromethyl)benzene (**2b**) (0.23 mL, 1.75 mmol) in THF (5 mL) at −78 °C. The mixture was maintained at −78 °C for 30 min, before being allowed to warm to room temperature overnight. The mixture was recooled to −78 °C, benzyl bromide (0.48 mL, 4.00 mmol) was added, and the mixture was stirred at −78 °C for 6 h. The mixture was warmed to room temperature and H_2O (5 mL) was added and the mixture extracted with EtOAc (15 mL × 3). The combined organic layers were washed with brine (5 mL), dried ($MgSO_4$), filtered and rotary evaporated. The residue was chromatographed (EtOAc:hexanes 1:20) to give **7b** (0.220 g, >98:2 dr, 67%) as a yellow solid. mp 31–33 °C (EtOAc/hexanes); R_f 0.71 (EtOAc:hexanes 1:3); [α]$_D^{25}$ = −46.6 (*c* 1.5, $CHCl_3$); IR 1689 (C=N), 1436, 1328, 1117 cm^{-1}; ^{1}H NMR (400 MHz, $CDCl_3$) δ 7.88 (m, 2H, Ar-H), 7.52 (m, 2H, Ar-H), 7.25 (m, 3H, Ar-H), 7.13 (m, 2H, Ar-H), 3.83 (s, 3H, OMe), 3.75 (d, 1H, *J* = 2.7 Hz, C<u>H</u>-iPr), 3.72 (s, 3H, OMe), 3.15 (AB quartet, 2H, *J* = 8.1 Hz, CH_2Ph), 2.12 (m, 1H, iPr), 0.91 (d, 3H, *J* = 6.9 Hz, iPr), 0.54 (d, 3H, *J* = 6.8 Hz, iPr); ^{13}C NMR (125 MHz, $CDCl_3$) δ 163.8, 161.0, 158.8, 145.2, 136.5, 130.5, 130.4, 128.2, 127.7, 126.6, 125.8 (q, $J_{C\text{-}F}$ = 245 Hz), 124.0, 123.8, 64.8, 60.4, 52.5, 52.3, 46.9, 31.1, 19.3, 17.0; ^{19}F NMR (200 MHz, $CDCl_3$) δ −62.8; HRMS (ESI) calcd. $C_{23}H_{26}F_3N_2O_2$: $(M + H)^+$, 419.1946; found: $(M + H)^+$, 419.1942.

(2*R*,5*R*)-2-Allyl-5-isopropyl-3,6-dimethoxy-2-(3-methoxyphenyl)-2,5-dihydropyrazine (7c)

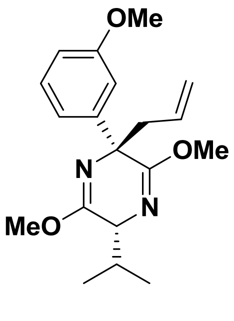

7c

sec-BuLi (in cyclohexane 1.1 M; 2.73 mL, 3.00 mmol) was added dropwise to imidate **1** (0.18 mL, 1.00 mmol) and 3-chloroanisole **2c** (0.21 mL, 1.75 mmol) in THF (5 mL) at −95 °C. The mixture was maintained at −95 °C for 30 min, before being allowed to warm to room temperature overnight. The mixture was recooled to −78 °C before allyl bromide (0.35 mL, 4.00 mmol) was added, and the mixture was stirred at −78 °C for 6 h. The mixture was warmed to room temperature and H_2O (5 mL) was added and the mixture extracted with EtOAc (15 mL × 3). The combined organic layers were washed with brine (5 mL), dried ($MgSO_4$), filtered and rotary evaporated. The residue was chromatographed (CH_2Cl_2:hexanes 1:1) to give **7c** (0.240 g, >98:2 dr,76%) as a colourless oil; R_f 0.69 (EtOAc:hexanes 1:3); $[\alpha]_D^{25}$ = −101.6 (*c* 0.9, $CHCl_3$); IR 1689 (C=N), 1602, 1434, 1236, 1142 cm^{-1}; ^{1}H NMR (400 MHz, $CDCl_3$) δ 7.25 (t, 1H, *J* = 8.0 Hz, Ar-H), 7.15 (dd, 1H, *J* = 7.9, 0.9 Hz, Ar-H), 7.11 (t, 1H, *J* = 2.0 Hz, Ar-H), 6.81 (dd, 1H, *J* = 8.1, 2.0 Hz, Ar-H), 5.64 (m, 1H, $C\underline{H}=CH_2$), 5.09 (m, 2H, $CH=C\underline{H_2}$), 3.96 (d, 1H, *J* = 4.1 Hz, $C\underline{H}$-iPr), 3.82 (s, 3H, OMe), 3.81 (s, 3H, OMe), 3.71 (s, 3H, OMe), 3.03 (dd, 1H, *J* = 13.2, 7.4 Hz, $C\underline{H}_2CH=CH_2$), 2.64 (dd, 1H, *J* = 13.1, 7.1 Hz, $C\underline{H}_2CH=CH_2$), 2.17 (m, 1H, iPr), 1.03 (d, 3H, *J* = 6.9 Hz, iPr), 0.66 (d, 3H, *J* = 6.8 Hz, iPr); ^{13}C NMR (125 MHz, $CDCl_3$) δ 163.5, 162.8, 159.1, 145.7, 133.7, 128.7, 119.1, 118.4, 113.0, 111.9, 64.1, 61.2, 55.1, 52.5, 52.5, 46.0, 31.7, 19.6, 17.7; HRMS (ESI) calcd. $C_{19}H_{27}N_2O_3$: $(M + H)^+$, 331.2022; found: $(M + H)^+$, 331.2015.

3. Hydrolysis products

(*R*)-Methyl 2-((*R*)-2-(*tert*-butoxycarbonylamino)-2-(2,5-dimethoxyphenyl)propanamido)-3-methylbutanoate (8a)

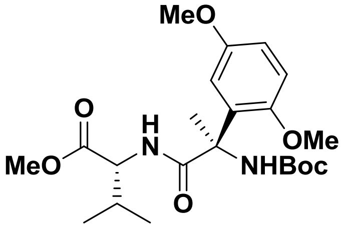

8a

0.5 M HCl (1.9 mL, 0.93 mmol) was added to imidate **6a** (0.138 g, 0.41 mmol) in THF (3 mL), and the resulting mixture was stirred at room temperature for 36 h. Saturated aqueous Na_2CO_3 solution was added dropwise until pH 9. The mixture was poured into H_2O (5 mL), and extracted with EtOAc (3 × 5 mL). The combined organic layers were washed with brine (5 mL), dried ($MgSO_4$),

filtered and rotary evaporated. The residue was redissolved in CH_2Cl_2 (3 mL), and di-isopropylethylamine (0.16 mL, 0.90 mmol) and di-*t*-butyl dicarbonate (0.19 g, 0.86 mmol) were added sequentially. The mixture was stirred at room temperature for 48 h, after which the mixture was rotary evaporated. The residue was chromatographed (CH_2Cl_2:MeOH (NH_3) 30:1) to give **8a** (0.134 g, 75%, 96:4 dr) as a yellow gum; R_f 0.74 (CH_2Cl_2:MeOH 10:1); $[\alpha]_D^{25}$ = −12.2 (*c* 0.8, $CHCl_3$); IR 3429 (NH), 1731 (C=O),1688 (C=O), 1464, 1223, 1168, 1143 cm^{-1}; ^{1}H NMR (400 MHz, $CDCl_3$) δ 7.11 (d, 1H, *J* = 2.3 Hz, Ar-H), 6.85 (m, 2H, Ar-H), 6.28 (br s, 2H, 2 × NH), 4.52 (dd, 1H, *J* = 8.7, 5.1 Hz, CH-iPr), 3.82 (s, 3H, OMe), 3.72 (s, 3H, OMe), 3.67 (s, 3H, OMe), 2.11 (m, 1H, iPr), 1.96 (s, 3H, CH_3), 1.35 (s, 9H, boc), 0.92 (d, 3H, *J* = 6.8 Hz, iPr), 0.82 (d, 3H, *J* = 6.9 Hz, iPr); ^{13}C NMR (125 MHz, $CDCl_3$) δ 174.3, 172.0, 154.2, 153.1, 151.0, 129.6, 115.6, 113.2, 112.2, 79.1, 60.4, 57.2, 55.8, 55.6, 52.0, 31.4, 28.3, 24.3, 19.0, 17.6; HRMS (CI) calcd. $C_{22}H_{34}N_2O_7Na$: $(M + Na)^+$, 461.2264; found: $(M + Na)^+$, 461.2204.

(*R*)-Methyl 2-((*S*)-2-(*tert*-butoxycarbonylamino)-2-(2,5-dimethoxyphenyl)-3-methoxypropanamido)-3-methylbutanoate (8d)

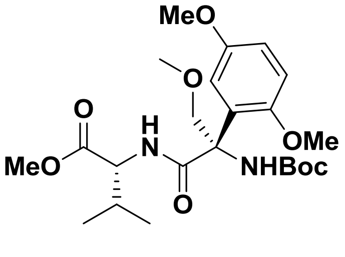

8d

0.5 M HCl (0.62 mL, 0.31 mmol) was added to imidate **6d** (0.050 g, 0.14 mmol) in THF (1 mL), and the resulting mixture was stirred at room temperature for 36 h. Saturated aqueous Na_2CO_3 solution was added dropwise until pH 9. The mixture was poured into H_2O (5 mL), and extracted with EtOAc (3 × 5 mL). The combined organic layers were washed with brine (5 mL), dried ($MgSO_4$), filtered and rotary evaporated. The residue was redissolved in CH_2Cl_2 (2 mL), and di-isopropylethylamine (0.050 mL, 0.31 mmol) and di-*t*-butyl dicarbonate (0.064 g, 0.29 mmol) were added sequentially. The mixture was stirred at room temperature for 48 h, after which the mixture was rotary evaporated. The residue was chromatographed (CH_2Cl_2:MeOH (NH_3) 50:1) to give **8d** (0.040 g, 61%, 95:5 dr) as a colourless oil. R_f 0.19 (CH_2Cl_2:MeOH 20:1); $[\alpha]_D^{25}$ = −1.3 (*c* 1.05, $CHCl_3$); IR 3412 (NH), 1743 (C=O), 1716 (C=O),1682 (C=O), 1496, 1467, 1229, 1169, 1052 cm^{-1}; ^{1}H NMR (400 MHz, $CDCl_3$) δ 7.05 (d, 1H, *J* = 2.52 Hz, Ar-H), 6.83 (m, 2H, Ar-H), 6.33 (br s, 1H, NH), 4.56 (dd, 1H, *J* = 8.8, 5.0 Hz, CHiPr), 4.39 (d, 1H, *J* = 9.3 Hz, CH_2OMe), 3.92 (br s, 1H, CH_2OMe), 3.80 (s, 3H, OMe), 3.72 (s, 3H, OMe), 3.66 (s, 3H, OMe), 3.42 (s, 3H, OMe), 2.13 (m, 1H, iPr), 1.37 (s, 9H, boc), 0.93 (d, 3H, *J* = 6.8 Hz, iPr), 0.84 (d, 3H, *J* = 6.9 Hz, iPr); ^{13}C NMR (125 MHz, $CDCl_3$) δ 172.0, 171.4, 154.4,

153.1, 151.4, 126.7, 116.0, 113.5, 112.2, 79.2, 73.5, 63.3, 59.0, 57.6, 55.9, 55.5, 51.9, 31.3, 28.3, 18.9, 17.5; HRMS (ESI) calcd. $C_{23}H_{36}N_2O_8Na$: $(M + Na)^+$, 491.2369; found: $(M + Na)^+$, 491.2351.

(*R*)-Methyl 2-((*R*)-2-(*tert*-butoxycarbonylamino)-2-(2,5-dimethoxyphenyl)pent-4-ynamido)-3-methylbutanoate (8e)

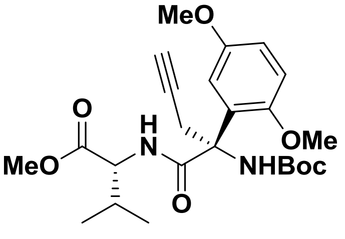

8e

0.5 M HCl (0.40 mL, 0.20 mmol) was added to imidate **6e** (0.032 g, 0.089 mmol) in THF (1 mL), and the resulting mixture was stirred at room temperature for 36 h. Saturated aqueous Na_2CO_3 solution was added dropwise until pH 9. The mixture was poured into H_2O (5 mL), and extracted with EtOAc (3 × 5 mL). The combined organic layers were washed with brine (5 mL), dried ($MgSO_4$), filtered and rotary evaporated. The residue was redissolved in CH_2Cl_2 (1.5 mL), and di*iso*propylethylamine (0.034 mL, 0.20 mmol) and di-*t*-butyl dicarbonate (0.041 g, 0.19 mmol) were added sequentially. The mixture was stirred at room temperature for 48 h, after which the mixture was rotary evaporated. The residue was purified by flash column chromatography (CH_2Cl_2:MeOH (NH_3) 50:1) to give **8e** (0.032 g, 72%, >98:2 dr) as a colourless oil. R_f 0.24 (CH_2Cl_2:MeOH 20:1); $[\alpha]_D^{25}$ = −12.4 (*c* 0.7, $CHCl_3$); IR 3405 (NH), 1740 (C=O), 1712 (C=O),1682 (C=O), 1496, 1468, 1226 cm^{-1}; ^1H NMR (400 MHz, $CDCl_3$) δ 7.13 (s, 1H, Ar-H), 7.00 (br s, 1H, NH), 6.86 (m, 2H, Ar-H), 6.48 (br s, 1H, NH), 4.55 (dd, 1H, *J* = 8.3, 4.9 Hz, CHiPr), 3.80 (s, 3H, OMe), 3.77 (s, 3H, OMe), 3.67 (s, 3H, OMe), 3.60 (br d, 1H, *J* = 15.7 Hz, C$\underline{H}_2$C≡CH), 3.40 (br d, 1H, *J* = 14.8 Hz, C$\underline{H}_2$C≡CH), 2.15 (m, 1H, iPr), 2.08 (s, 1H, C≡C$\underline{H}$), 1.42 (s, 9H, boc), 0.95 (d, 3H, *J* = 6.9 Hz, iPr), 0.87 (d, 3H, *J* = 6.9 Hz, iPr); ^{13}C NMR (125 MHz, $CDCl_3$) δ 171.9, 171.3, 154.1, 153.2, 150.8, 127.1, 115.4, 114.1, 112.5, 79.9, 79.5, 72.0, 62.9, 57.7, 55.9, 55.6, 51.9, 31.4, 28.3, 26.2, 19.0, 17.5; HRMS (ESI) calcd. $C_{24}H_{34}N_2O_7Na$: $(M + Na)^+$, 485.2264; found: $(M + Na)^+$, 485.2246.

(*R*)-Methyl 2-((*R*)-2-amino-2-(3-methoxyphenyl)pent-4-enamido)-3-methylbutanoate (9)

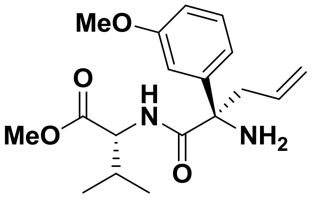

9

0.5 M HCl (0.69 mL, 0.34 mmol) was added to imidate **7c** (0.051 g, 0.15 mmol) in THF (1.5 mL), and the resulting mixture was stirred at room temperature for 36 h. Saturated

aqueous Na_2CO_3 solution was added dropwise until pH 9. The mixture was poured into H_2O (5 mL), and extracted with EtOAc (3 × 5 mL). The combined organic layers were washed with brine (5 mL), dried ($MgSO_4$), filtered and rotary evaporated. The residue was chromatographed (EtOAc:hexanes 1:5) to give **9** (0.020 g, 40%) as a colourless oil. R_f 0.54 (EtOAc:hexanes 1:1); $[\alpha]_D^{25}$ = 3.3 (*c* 1.6, $CHCl_3$); IR 3367 (NH), 1742 (C=O), 1675 (C=O), 1493, 1438, 1259 cm^{-1}; ^1H NMR (400 MHz, $CDCl_3$) δ 8.10 (d, 1H, *J* = 9.0 Hz, NH), 7.29 (m, 1H, Ar-H), 7.19 (dd, 2H, *J* = 7.8, 1.0 Hz, Ar-H), 6.84 (m, 1H, Ar-H), 5.70 (m, 1H, C$\underline{H}$=CH_2), 5.22 (m, 2H, C$\underline{H}_2$CH=CH_2), 4.45 (dd, 1H, *J* = 9.1, 4.9 Hz, C$\underline{H}$iPr), 3.83 (s, 3H, OMe), 3.77 (s, 3H, OMe), 3.20 (dd, 1H, *J* = 13.5, 6.3 Hz, CH=C$\underline{H}_2$), 2.71 (dd, 1H, *J* = 13.4, 8.3 Hz, CH=C$\underline{H}_2$), 2.15 (m, 1H, iPr), 1.80 (br s, 2H, NH_2), 0.83 (d, 3H, *J* 6.9 Hz, iPr), 0.80 (d, 3H, *J* = 6.9 Hz, iPr); ^{13}C NMR (125 MHz, $CDCl_3$) δ 174.2, 172.5, 159.7, 144.5, 133.6, 129.4, 120.1, 117.8, 112.8, 111.6, 62.3, 57.0, 55.2, 52.0, 44.9, 31.2, 18.9, 17.5; HRMS (ESI) calcd. $C_{18}H_{27}N_2O_4$: $(M + H)^+$, 335.1971; found: $(M + H)^+$, 335.1960.

(*R*)-Methyl 2-amino-2-(3-methoxyphenyl)pent-4-enoate (7c)

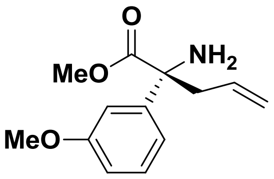

10

Imidate **7c** (0.024 g, 0.073 mmol) was stirred in 6 M H_2SO_4 (1 mL) at room temperature for 3 days. Saturated aqueous Na_2CO_3 solution was added dropwise until pH 9. The mixture was poured into H_2O (5 mL), and extracted with CH_2Cl_2 (3 × 5 mL). The combined organic layers were washed with brine (5 mL), dried ($MgSO_4$), filtered and rotary evaporated. The residue was chromatographed (EtOAc:hexanes 1:3) to give **10** (0.010 g, 58%, 90% ee) as a colourless oil. R_f 0.65 (EtOAc:hexanes 1:1); $[\alpha]_D^{25}$ = 12.1 (*c* 1.4, $CHCl_3$); IR 2963 (NH), 1731 (C=O), 1493, 1438, 1259 cm^{-1}; ^1H NMR (400 MHz, $CDCl_3$) δ 7.30 (m, 1H, Ar-H), 7.14 (m, 2H, Ar-H), 6.89 (dd, 1H, *J* = 7.8, 2.3 Hz, Ar-H), 5.72 (m, 1H, C$\underline{H}$=CH_2), 5.21 (m, 2H, C$\underline{H}_2$CH=CH_2), 3.85 (s, 3H, OMe), 3.76 (s, 3H, OMe), 3.00 (dd, 1H, *J* = 13.6, 6.7 Hz, C=C$\underline{H}_2$), 2.69 (dd, 1H, *J* = 13.7, 7.8 Hz, C=C$\underline{H}_2$), 1.91 (br s, 2H, NH_2); ^{13}C NMR (125 MHz, $CDCl_3$) δ 175.5, 159.7, 144.4, 132.9, 129.4, 120.0, 117.7, 112.7, 111.5, 63.1, 55.3, 52.6, 44.6; HRMS (ESI) calcd. $C_{13}H_{18}NO_3$: $(M + H)^+$, 236.1287; found: $(M + H)^+$, 236.1285. The enantiomeric excess was determined by HPLC. [CHIRALPACK® IC, 254 nm, hexane:isopropanol = 60:40, 1.0 mL/min]: 3.773 min (major), 15.399 min (minor).

(*R*)-Methyl 2-(*tert*-butoxycarbonylamino)-2-(2,5-dimethoxyphenyl)propanoate (11)

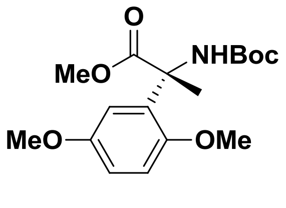

11

Imidate **6a** (0.10 g, 0.30 mmol) was stirred in 6 M H_2SO_4 (1 mL) at room temperature for 3 days. Saturated aqueous Na_2CO_3 solution was added dropwise until pH 9. The mixture was poured into H_2O (5 mL), and extracted with CH_2Cl_2 (3 × 5 mL). The combined organic layers were washed with brine (5 mL), dried ($MgSO_4$), filtered and rotary evaporated. The residue was redissolved in CH_2Cl_2 (2 mL), and sequentially diisopropylethylamine (0.11 mL, 0.66 mmol) and di-*t*-butyl dicarbonate (0.14 g, 0.63 mmol) were added. The mixture was stirred at room temperature for 48 h, after which the mixture was rotary evaporated. The residue was chromatographed (EtOAc:hexanes 1:5) to give **11** (0.057 g, 56%, 90% ee) as a colourless oil. R_f 0.54 (EtOAc:hexanes 1:3); $[\alpha]_D^{25}$ = −36.8 (*c* 0.6, $CHCl_3$); IR 3435 (NH), 1741 (C=O), 1717 (C=O), 1495, 1272 cm^{-1}; ^1H NMR (400 MHz, $CDCl_3$) δ 7.06 (s, 1H, Ar-H), 6.80 (br s, 2H, Ar-H), 6.14 (s, 1H, NH), 3.80 (s, 3H, OMe), 3.74 (s, 3H, OMe), 3.71 (s, 3H, OMe), 1.97 (s, 3H, CH_3), 1.33 (s, 9H, boc); ^{13}C NMR (125 MHz, $CDCl_3$) δ 174.9, 154.0, 153.2, 150.7, 130.6, 115.4, 112.6, 112.2, 79.2, 59.4, 56.2, 55.6, 52.8, 28.3, 22.6; HRMS (ESI) calcd. $C_{17}H_{25}NO_6Na$: $(M + Na)^+$, 362.1580; found: $(M + Na)^+$, 362.1563. The enantiomeric excess was determined by HPLC. [CHIRALPACK® IC, 254 nm, hexane:isopropanol = 90:10, 1.0 mL/min]: 8.533 min (major), 10.813 min (minor).

(3*R*,5*R*)-3-Amino-3-(2,5-dimethoxyphenyl)-5-methyldihydrofuran-2(3*H*)-one (12)

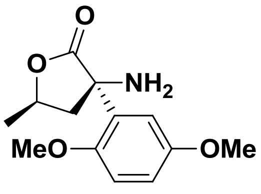

12

0.5 M HCl (0.23 mL, 0.11 mmol) was added to imidate **6j** (0.020 g, 0.050 mmol) in THF (0.5 mL), and the resulting mixture was stirred at room temperature for 48 h. Saturated aqueous Na_2CO_3 solution was added dropwise until pH 9. The mixture was poured into H_2O (5 mL), and extracted with EtOAc (3 × 5 mL). The combined organic layers were washed with brine (5 mL), dried ($MgSO_4$), filtered and rotary evaporated. The residue was chromatographed (CH_2Cl_2:MeOH (NH_3) 40:1) to give **12** (0.010 g, 80%, 96% ee) as a colourless oil. R_f 0.48 (CH_2Cl_2:MeOH (NH_3) 20:1); $[\alpha]_D^{25}$ = −13.6 (*c* 1.4, $CHCl_3$); IR 2932 (NH), 1760 (C=O), 1494, 1229 cm^{-1}; ^1H NMR (400 MHz, $CDCl_3$) δ 7.01 (d, 1H, J = 2.9 Hz, Ar-H), 6.89 (d, 1H, J = 8.8 Hz, Ar-H), 6.83 (dd, 1H, J = 8.9, 2.9 Hz, Ar-H), 4.52 (m, 1H, $C\underline{H}CH_3$), 3.86 (s, 3H, OMe), 3.80 (s, 3H, OMe), 2.90 (dd, 1H, J = 13.2,

6.4 Hz, C<u>H</u>$_2$), 2.04 (dd, 1H, *J* = 13.1, 8.4 Hz, C<u>H</u>$_2$), 1.84 (br s, 2H, NH_2), 1.49 (d, 3H, *J* 6.2 Hz, CH_3); ^{13}C NMR (125 MHz, $CDCl_3$) δ 179.5, 153.7, 150.4, 131.6, 113.3, 113.0, 112.5, 62.3, 60.4, 55.9, 55.8, 46.3, 14.2; HRMS (ESI) calcd. $C_{13}H_{18}NO_4$: (M + H)$^+$, 252.1236; found: (M + H)$^+$, 252.1231. The enantiomeric excess was determined by HPLC. [CHIRALPACK® IC, 254 nm, hexane:isopropanol = 60:40, 1.0 mL/min]: 11.146 min (major), 16.452 min (minor).

1-((2*R*,5*S*)-2-(2,5-Dimethoxyphenyl)-5-isopropyl-3,6-dimethoxy-2,5-dihydropyrazin-2-yl)ethanone (13)

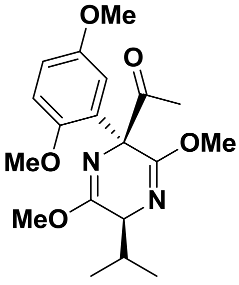

13

0.5 M HCl (0.55 mL, 0.27 mmol) was added to imidate **6g** (0.044 g, 0.12 mmol) in THF (2 mL), and the resulting mixture was stirred at room temperature for 36 h. Saturated aqueous Na_2CO_3 solution was added dropwise until pH 9. The mixture was poured into H_2O (5 mL), and extracted with EtOAc (3 × 5 mL). The combined organic layers were washed with brine (5 mL), dried ($MgSO_4$), filtered and rotary evaporated. The residue was chromatographed (EtOAc:hexanes 1:8) to give **13** (0.042 g, 95%, 88:12 dr) as a yellow oil. R_f 0.55 (EtOAc:hexanes 1:4); $[\alpha]_D^{25}$ = 13.7(*c* 1.0, $CHCl_3$); IR 1747 (C=O), 1681 (C=N), 1503, 1416, 1223, 1050 cm^{-1}; ^{1}H NMR (400 MHz, $CDCl_3$) δ 7.15 (d, 1H, *J* = 3.0 Hz, Ar-H), 6.87 (d, 1H, *J* = 8.9 Hz, Ar-H), 6.82 (dd, 1H, *J* = 8.9, 3.0 Hz, Ar-H), 4.30 (d, 1H, *J* = 10.5 Hz, C<u>H</u>-iPr), 3.82 (s, 3H, OMe), 3.79 (s, 3H, OMe), 3.77 (s, 3H, OMe), 3.63 (s, 3H, OMe), 2.74 (m, 1H, iPr), 2.40 (s, 3H, C<u>H$_3$</u>), 1.16 (d, 3H, *J* = 6.5 Hz, iPr), 0.82 (d, 3H, *J* = 6.8 Hz, iPr); ^{13}C NMR (125 MHz, $CDCl_3$) δ 169.7, 153.7, 151.2, 143.9, 137.4, 124.6, 116.3, 115.6, 113.9, 112.9, 62.4, 60.9, 56.6, 55.8, 52.4, 28.9, 20.9, 19.6, 19.0, 14.7; MS (ESI) calcd. $C_{19}H_{27}N_2O_5$: (M + H)$^+$, 363; found: (M + H)$^+$, 363.
