# Supplementary material for: Asymmetric synthesis of quaternary aryl amino acid derivatives via a three-component aryne coupling reaction
Source: Beilstein J Org Chem. 2011 Nov 25;7:1570–6. doi: 10.3762/bjoc.7.185 (PMC3252860; doi:10.3762/bjoc.7.185)
Supplement: File 2 — NMR spectral data for compounds 6a–j and 7b–c. [file Beilstein_J_Org_Chem-07-1570-s002.pdf]

Supporting Information File 2

for

Asymmetric synthesis of quaternary aryl amino acid derivatives via a three-component aryne coupling reaction

Elizabeth P. Jones[1], Peter Jones[2], Andrew J. P. White[1] and Anthony G. M. Barrett*[1]

Address: [1]Department of Chemistry, Imperial College London, London, SW7 2AZ, England and [2]Worldwide Medicinal Chemistry, Pfizer Limited, Ramsgate Road, Sandwich, Kent, CT13 9NJ, England

Email: Anthony G. M. Barrett*[1] - agm.barrett@imperial.ac.uk

* Corresponding author

NMR spectral data for compounds 6a–j and 7b–c.

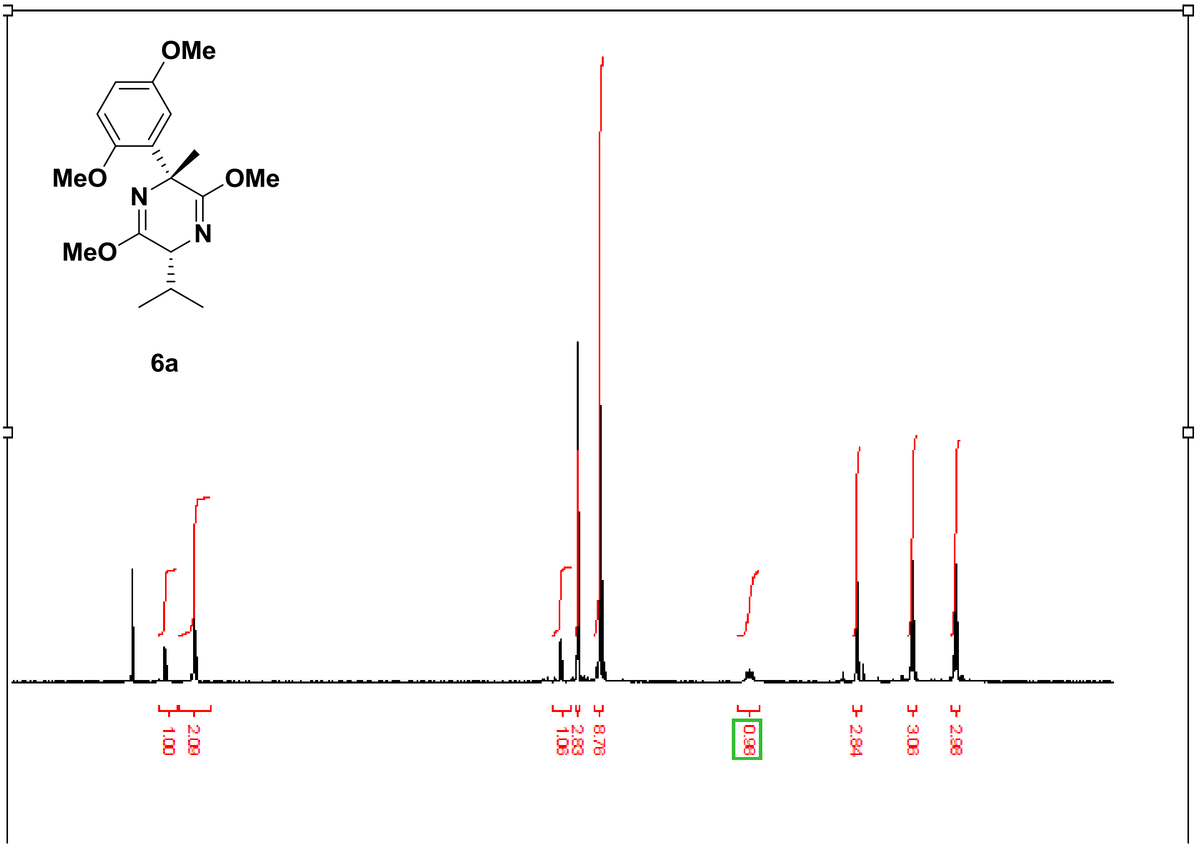
OMe
MeO
OMe
N
N
MeO
6a
1.00
2.09
1.06
2.83
8.76
0.98
2.84
3.06
2.98

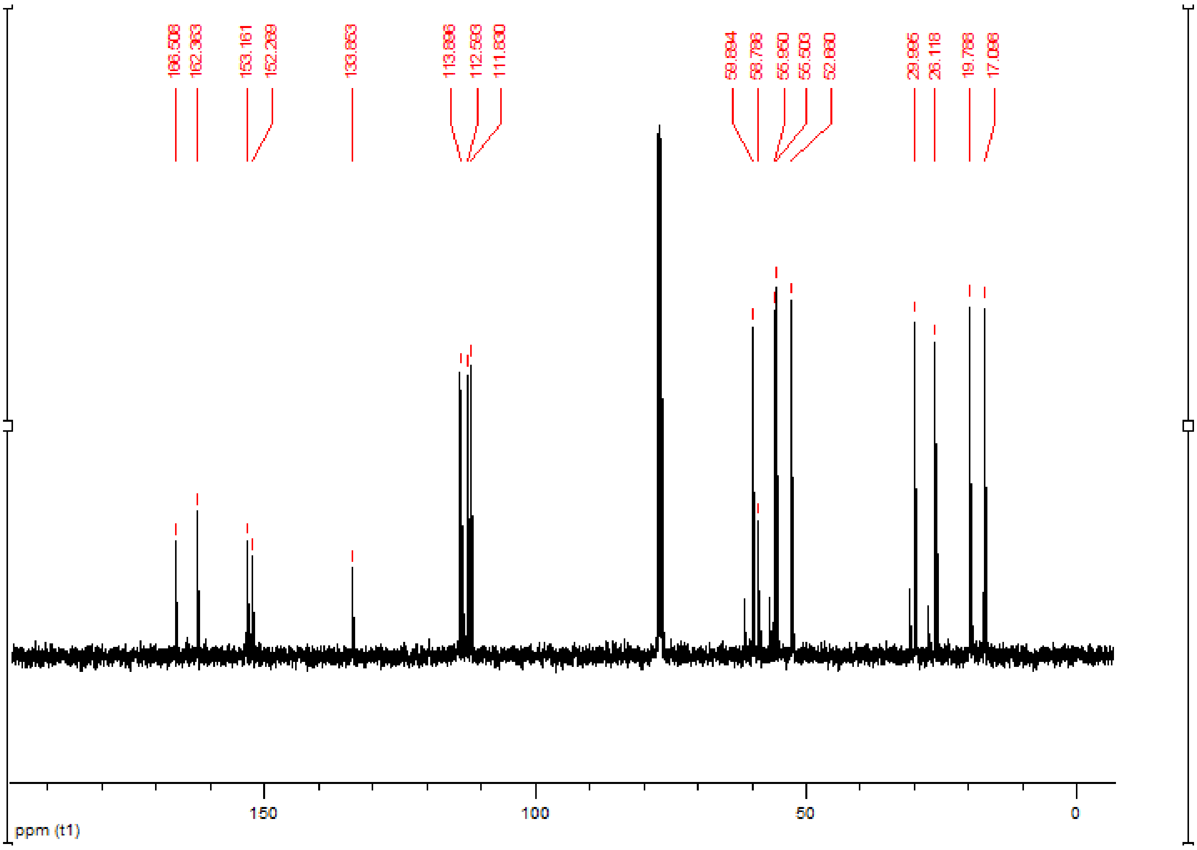
166.508
162.363
153.161
152.269
133.853
113.896
112.593
111.830
59.894
58.786
55.950
55.503
52.680
29.955
26.118
19.788
17.098
150
100
50
0
ppm (t1)

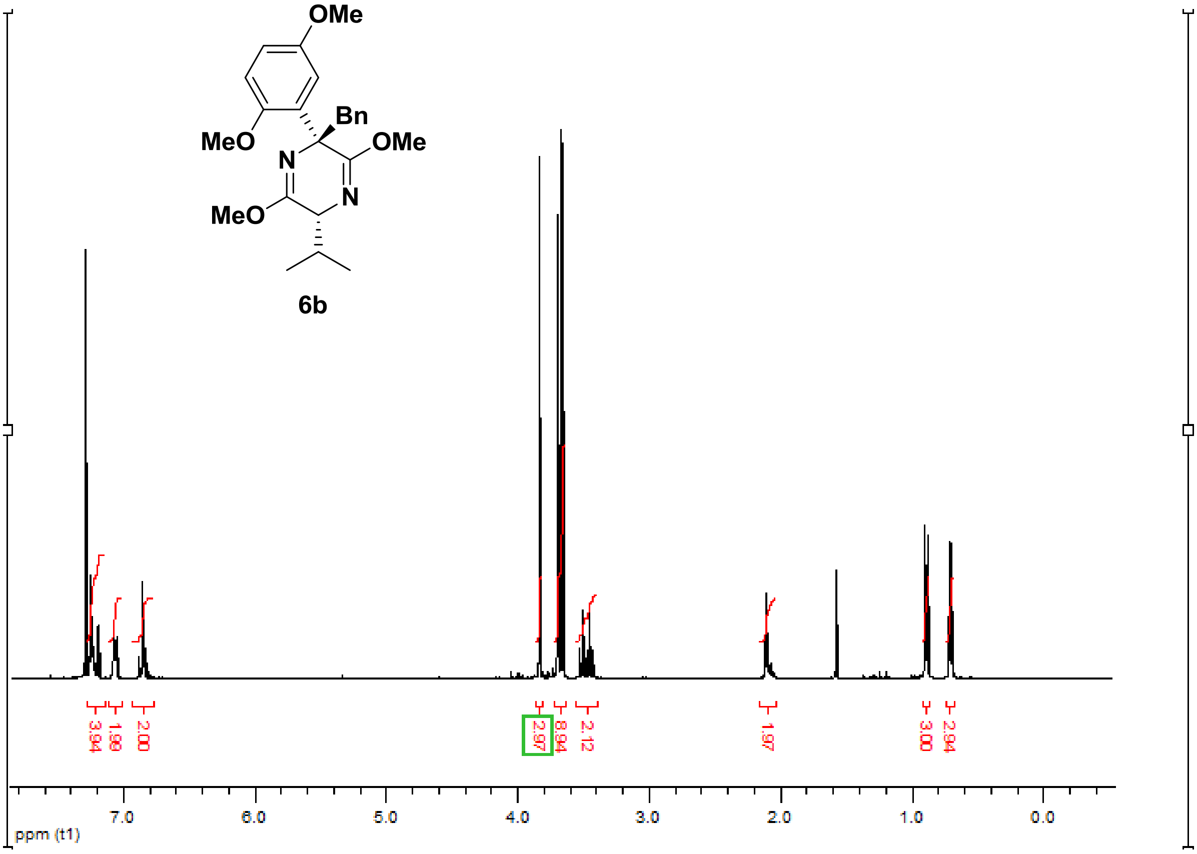
OMe
Bn
MeO
OMe
N
N
MeO
6b
3.94
1.99
2.00
2.97
8.94
2.12
1.97
3.00
2.94
7.0
6.0
5.0
4.0
3.0
2.0
1.0
0.0
ppm (t1)

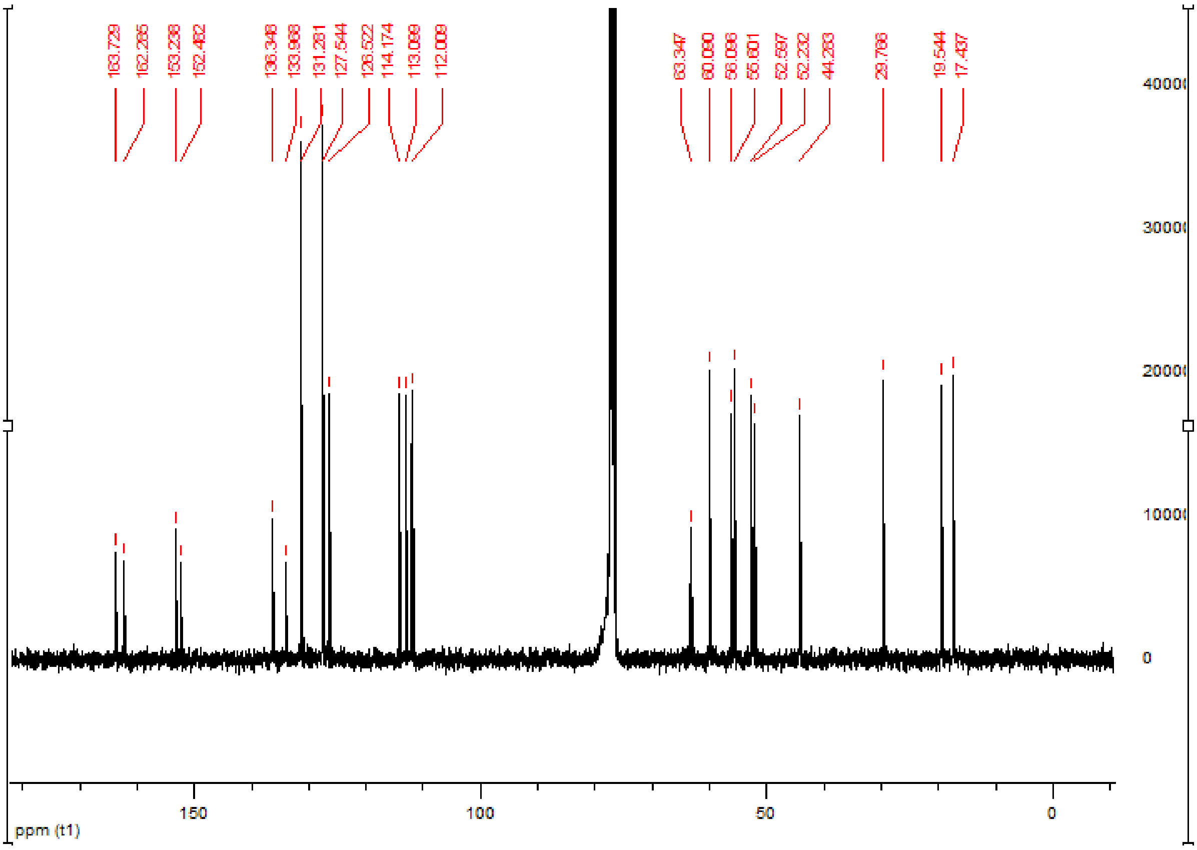
163.729
162.265
153.238
152.462
136.348
133.968
131.281
127.544
126.522
114.174
113.089
112.009
63.347
60.090
56.086
55.601
52.597
52.232
44.283
29.766
19.544
17.437
150
100
50
0
ppm (t1)

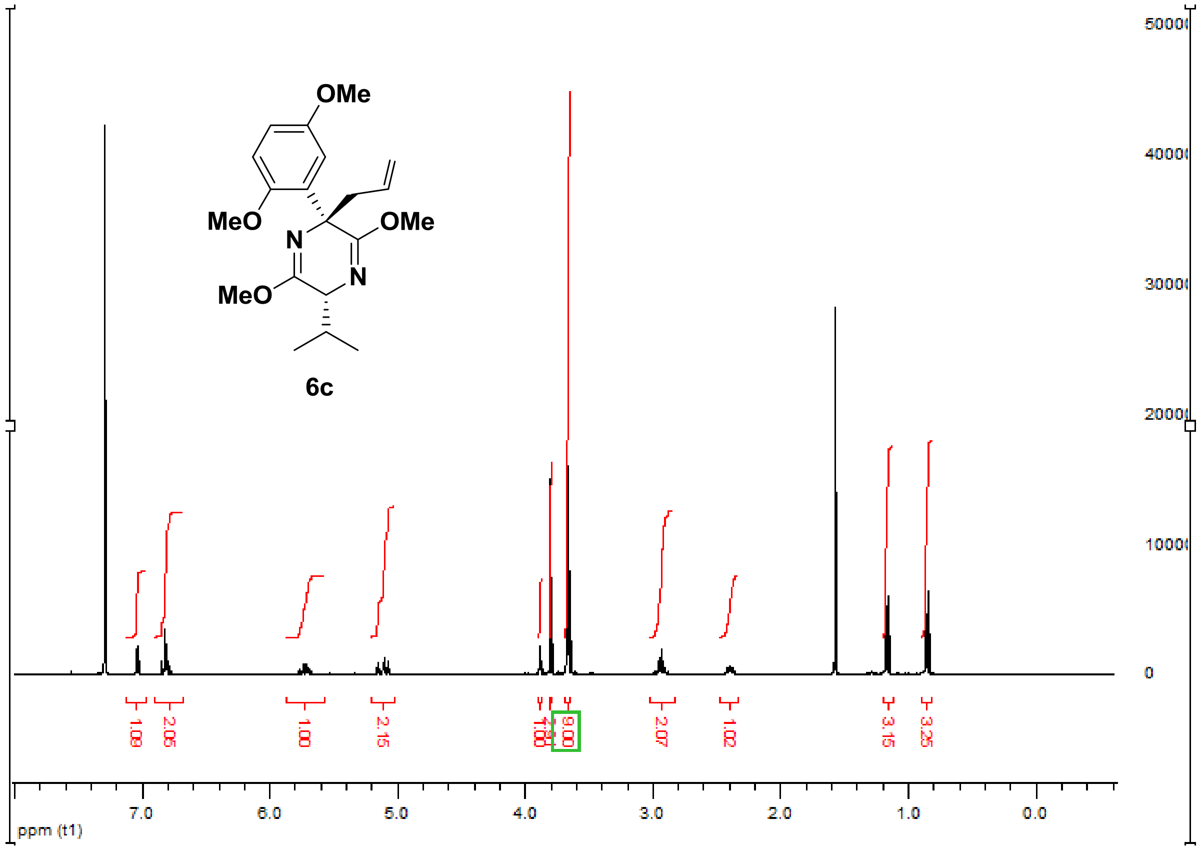
OMe
MeO
N
OMe
N
MeO
6c
1.09
2.06
1.00
2.15
1.00
9.00
2.07
1.02
3.15
3.25
7.0
6.0
5.0
4.0
3.0
2.0
1.0
0.0
ppm (t1)
0
10000
20000
30000
40000
50000

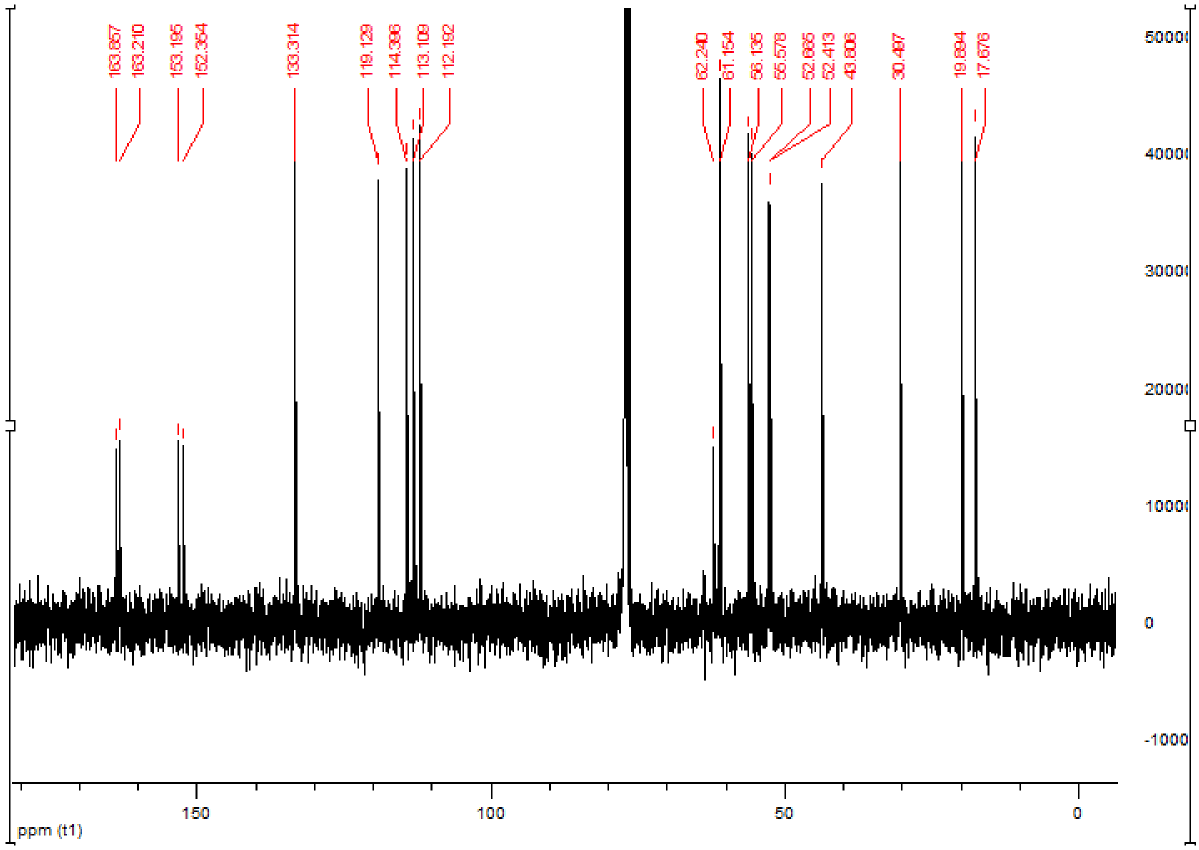
163.857
163.210
153.195
152.354
133.314
119.129
114.396
113.109
112.192
62.240
61.154
56.136
55.578
52.685
52.413
43.806
30.497
19.894
17.676
150
100
50
0
ppm (t1)
-1000
0
10000
20000
30000
40000
50000

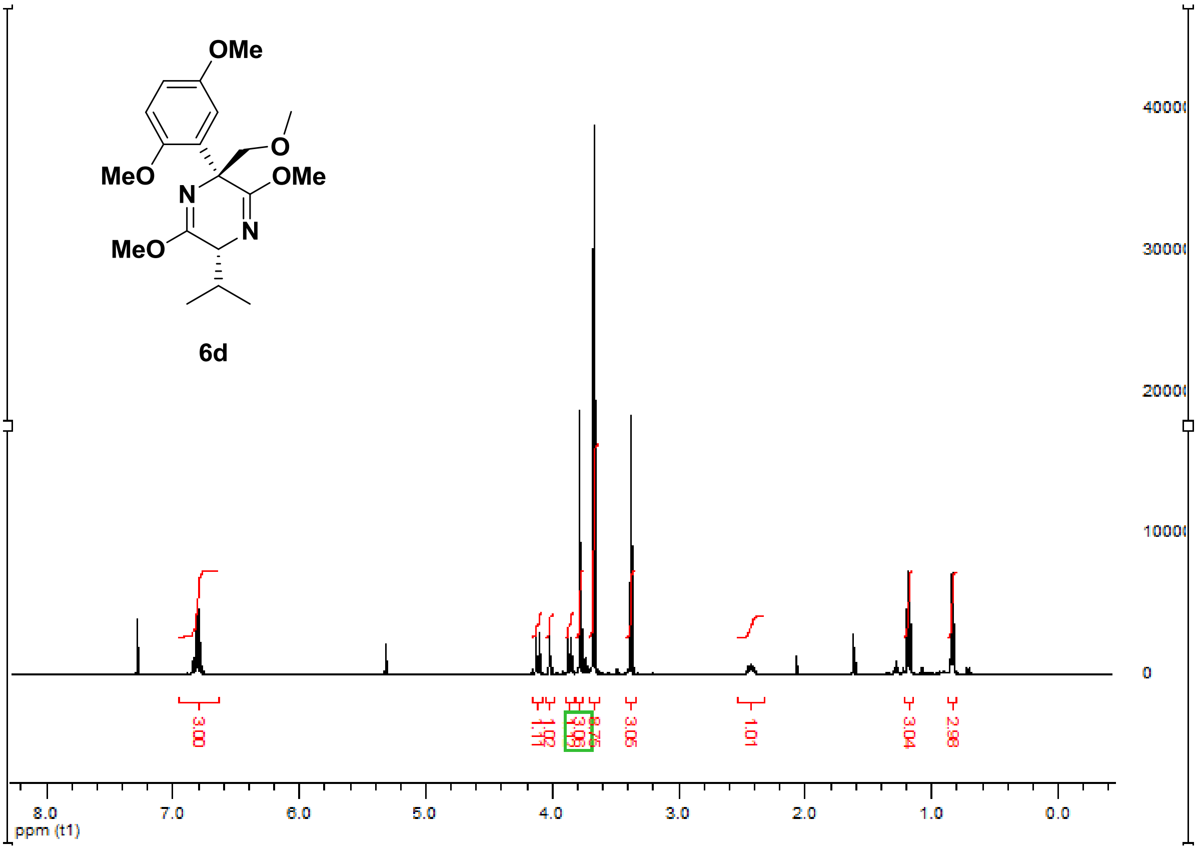
OMe
MeO
O
OMe
N
N
MeO
6d
3.00
1.01
3.05
3.04
2.98
8.0
7.0
6.0
5.0
4.0
3.0
2.0
1.0
0.0
ppm (t1)

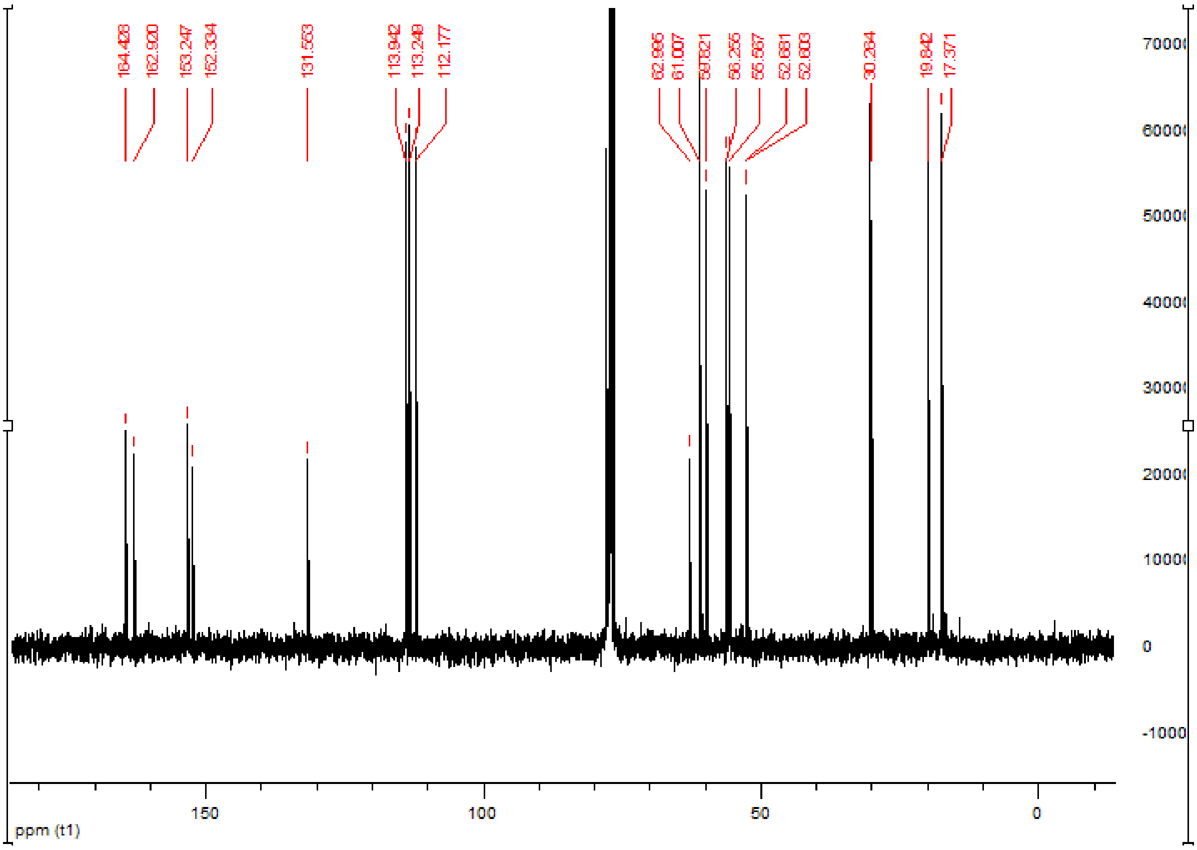
164.428
162.920
153.247
152.334
131.563
113.942
113.249
112.177
62.995
61.007
59.821
56.255
55.567
52.681
52.603
30.284
19.842
17.371
150
100
50
0
ppm (t1)

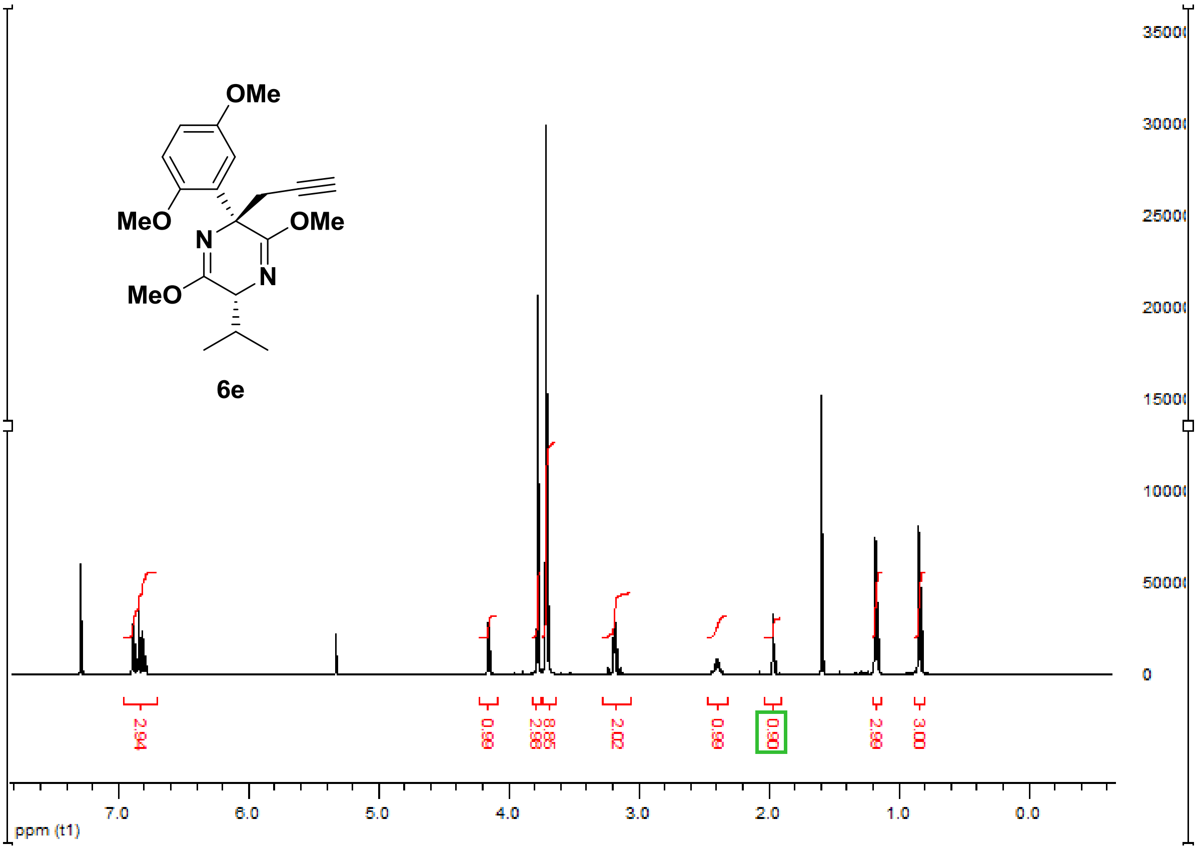
OMe
MeO
OMe
N
N
MeO
6e
2.94
0.99
2.98
8.85
2.02
0.99
0.90
2.99
3.00
ppm (t1)
7.0
6.0
5.0
4.0
3.0
2.0
1.0
0.0

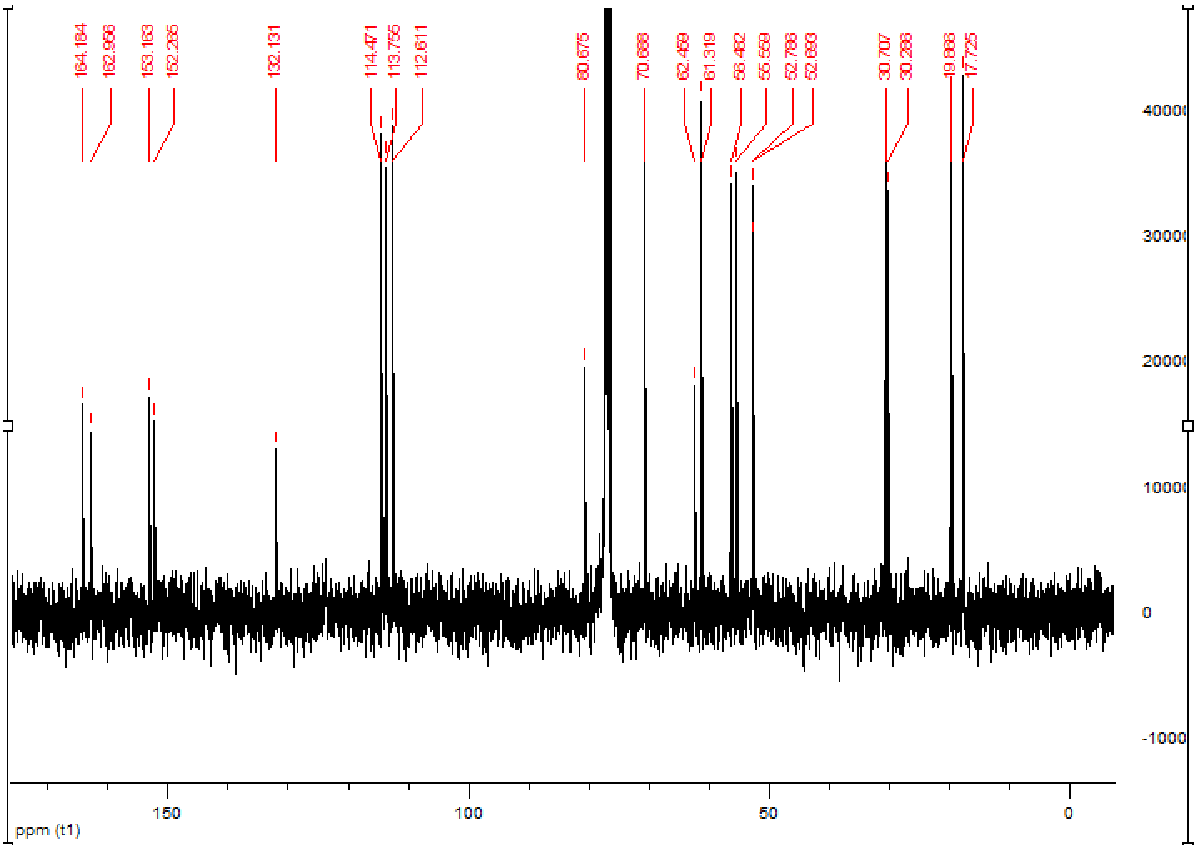
164.184
162.956
153.163
152.265
132.131
114.471
113.755
112.611
80.675
70.688
62.459
61.319
56.482
55.559
52.786
52.693
30.707
30.286
19.886
17.725
ppm (t1)
150
100
50
0

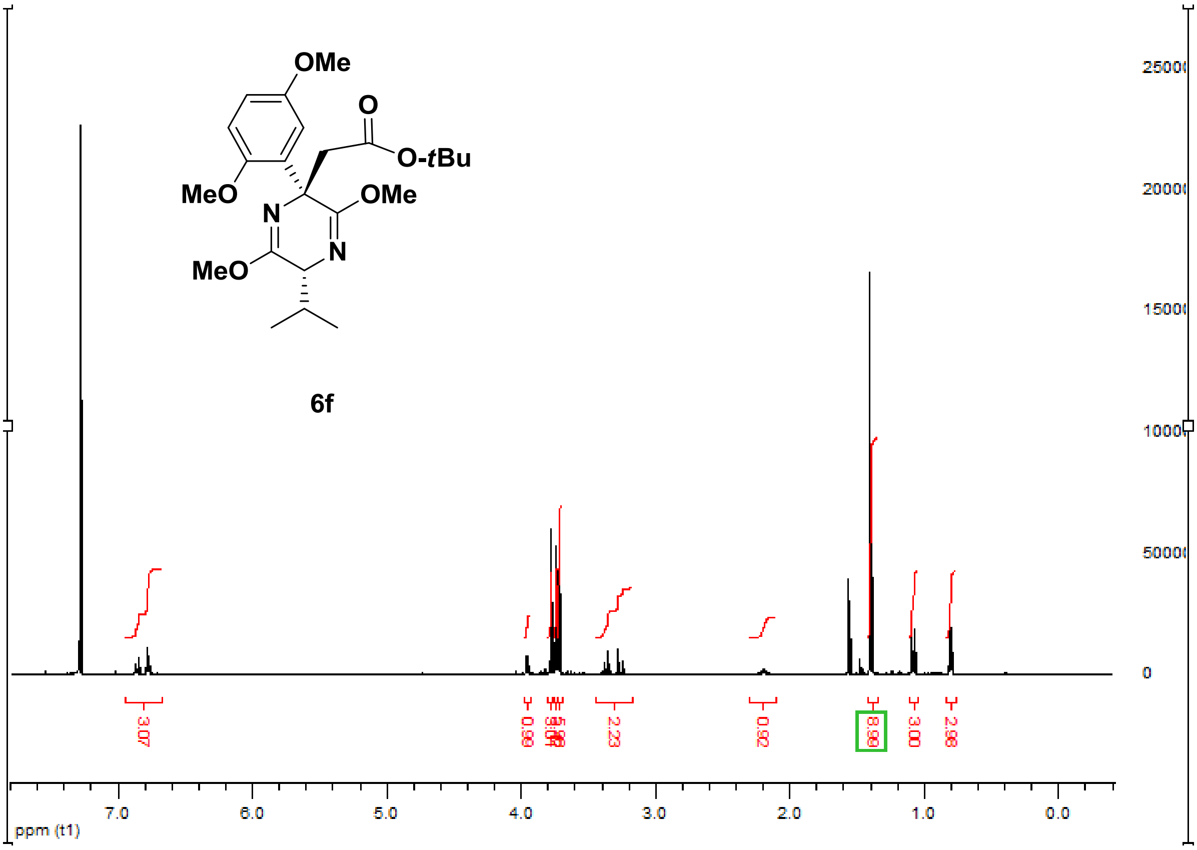
OMe
O
O-tBu
MeO
OMe
N
N
MeO
6f
3.07
0.99
2.23
0.92
3.00
2.96
7.0
6.0
5.0
4.0
3.0
2.0
1.0
0.0
ppm (t1)

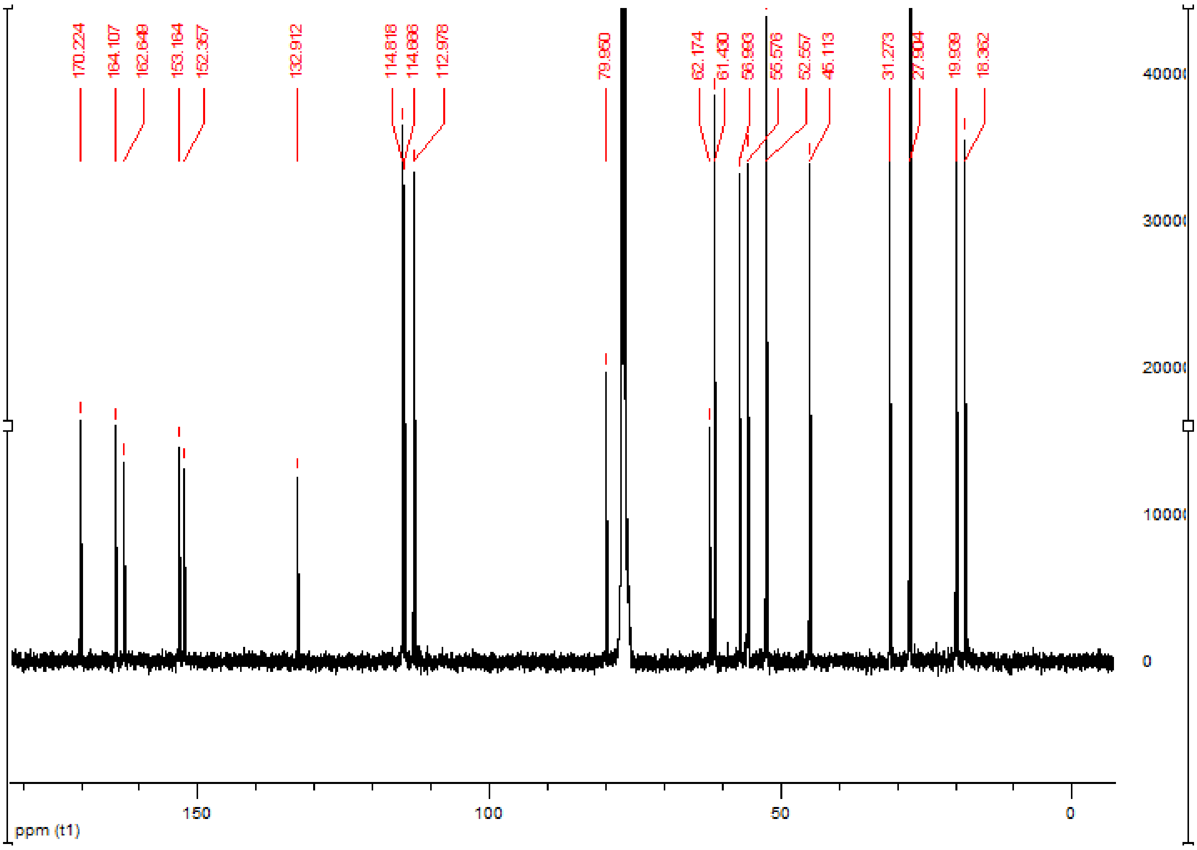
170.224
164.107
162.648
153.164
152.357
132.912
114.818
114.666
112.978
79.950
62.174
61.430
56.963
55.576
52.557
45.113
31.273
27.904
19.939
18.362
150
100
50
0
ppm (t1)

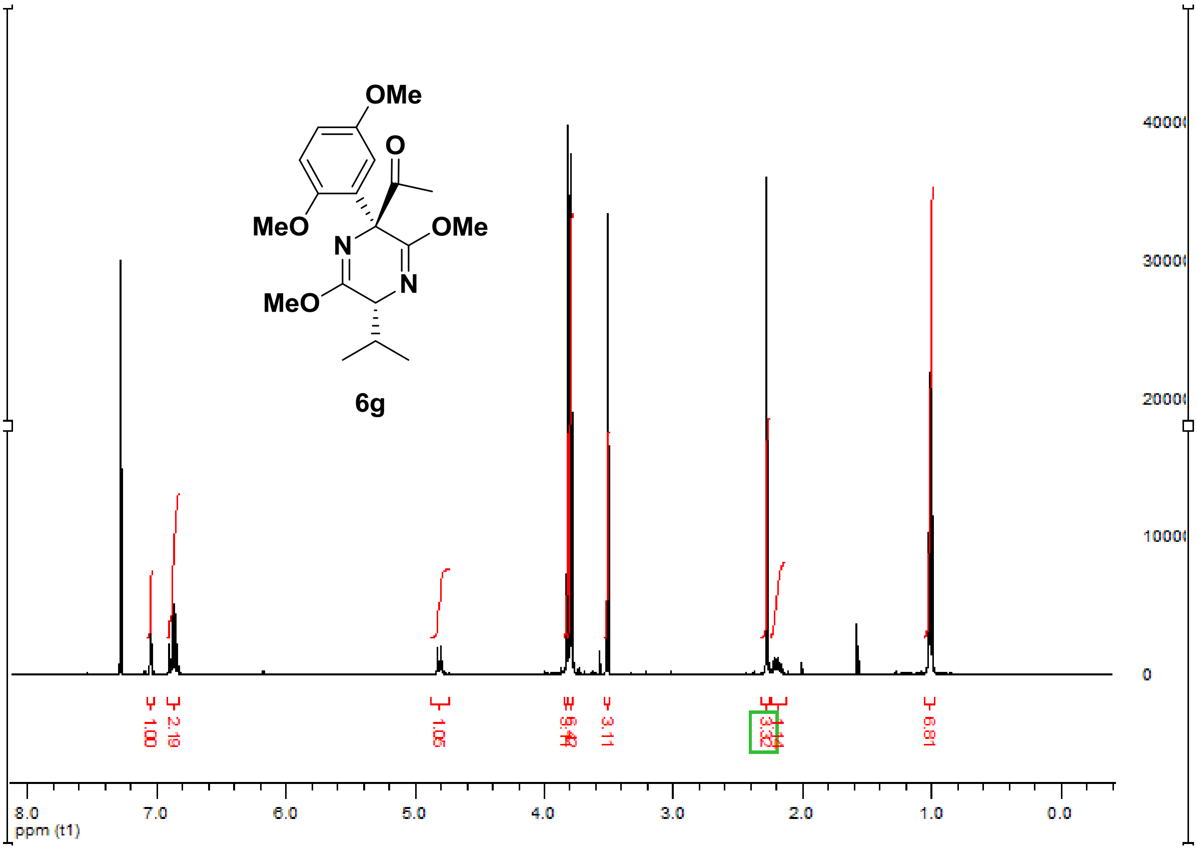
OMe
O
MeO
OMe
N
N
MeO
6g
1.00
2.19
1.05
3.11
6.81
8.0
7.0
6.0
5.0
4.0
3.0
2.0
1.0
0.0
ppm (t1)

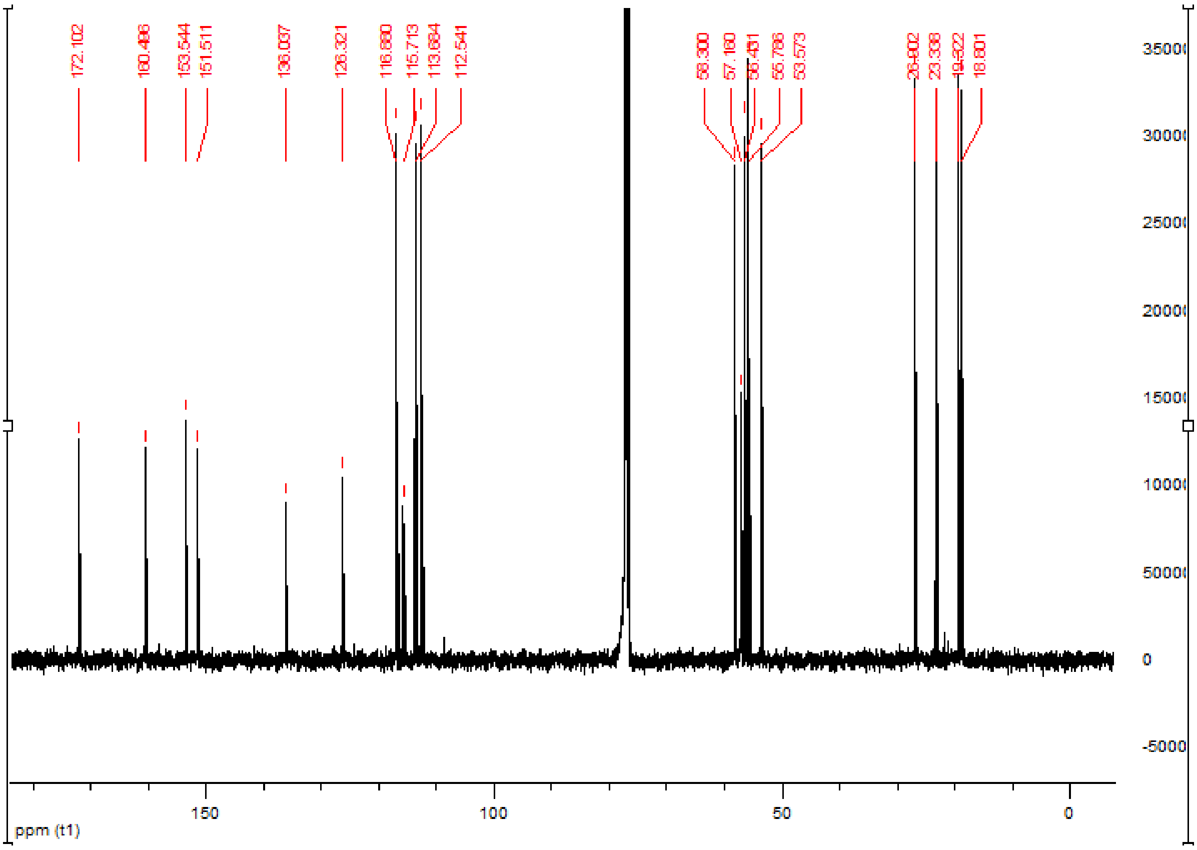
172.102
153.544
151.511
136.037
126.321
115.713
112.541
58.300
57.160
53.573
18.801
150
100
50
0
ppm (t1)

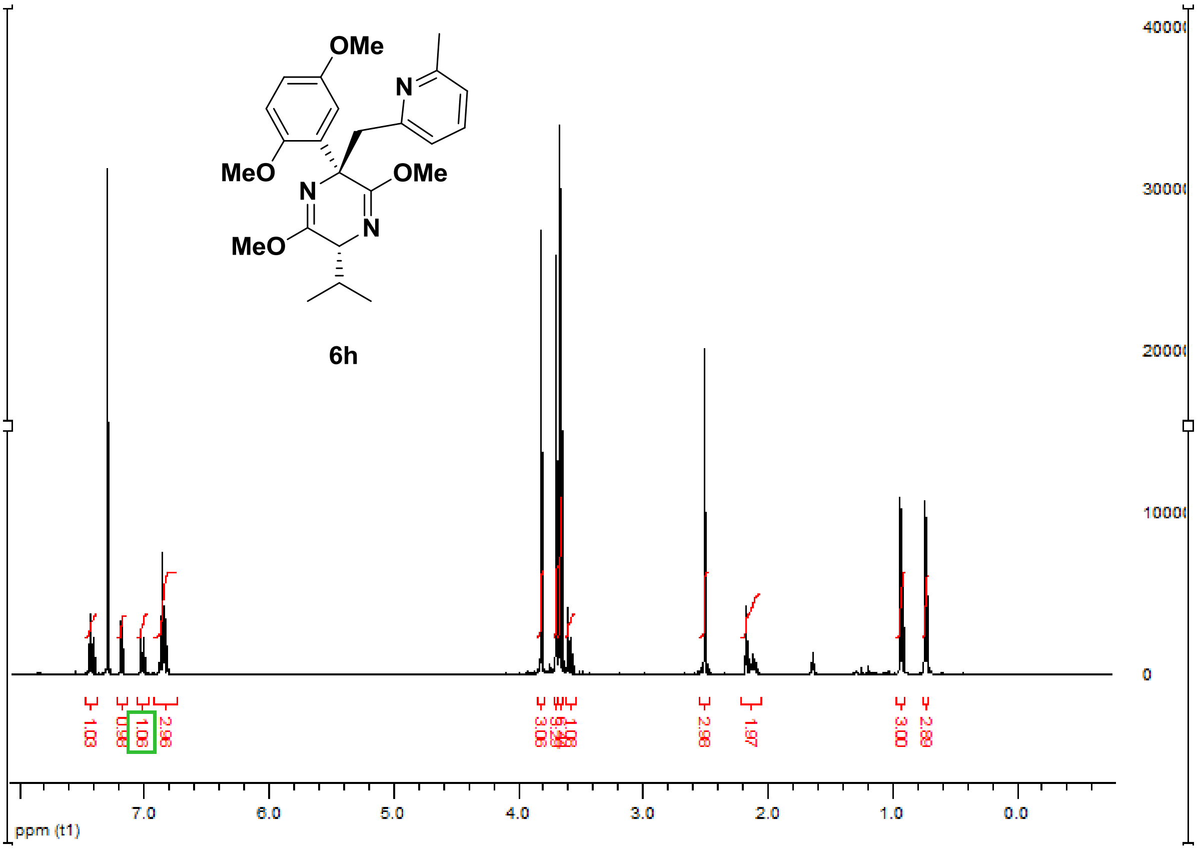

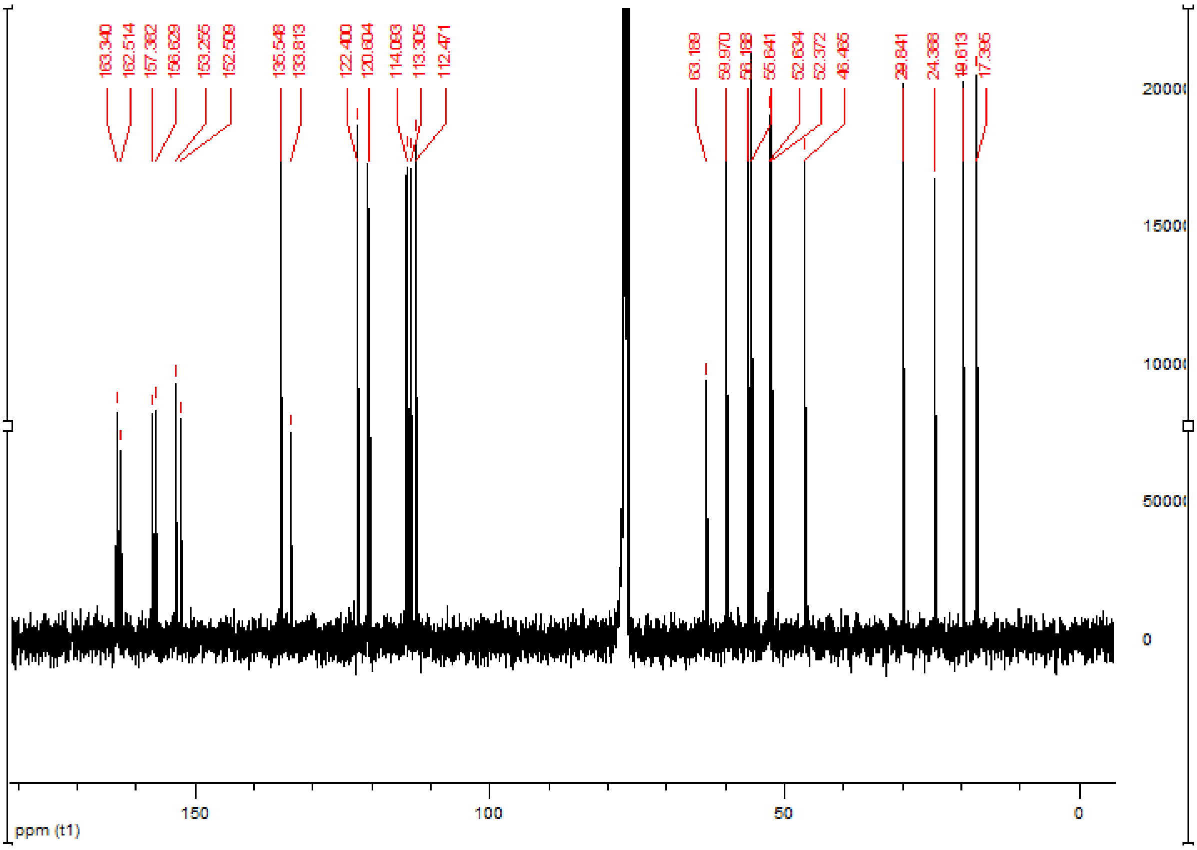

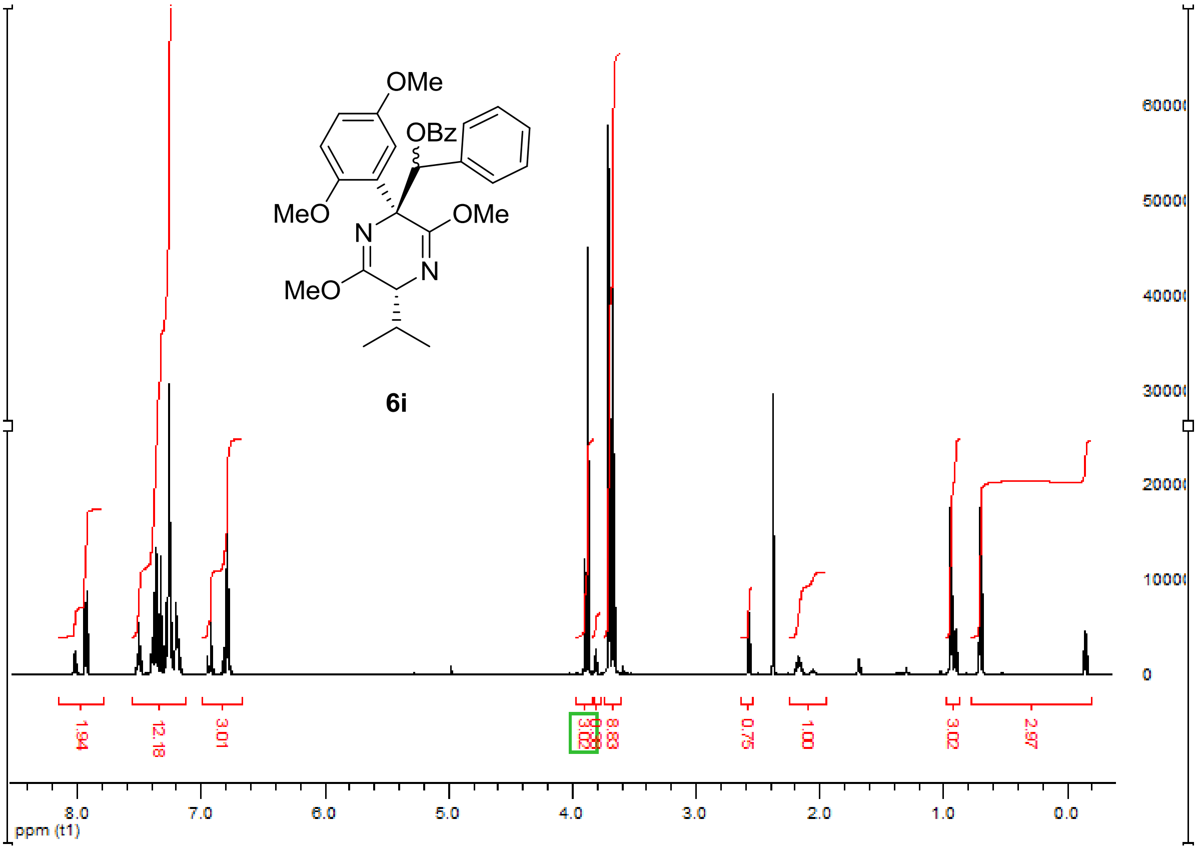
OMe
OBz
MeO
OMe
N
N
MeO
6i
1.94
12.18
3.01
3.02
8.83
0.75
1.00
3.02
2.97
8.0 7.0 6.0 5.0 4.0 3.0 2.0 1.0 0.0
ppm (t1)

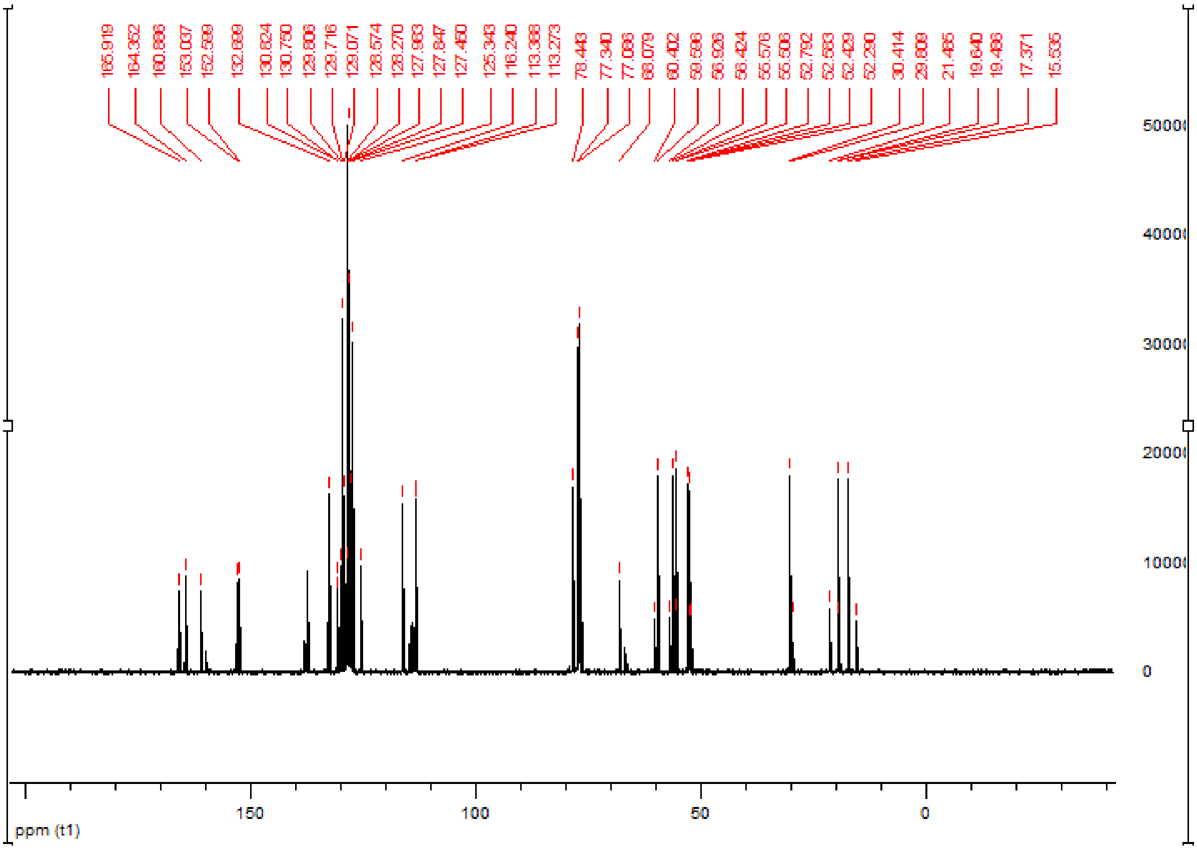
165.919
164.352
160.886
153.037
152.589
132.889
130.824
130.750
129.806
129.716
129.071
128.574
128.270
127.963
127.847
127.450
125.343
116.240
113.368
113.273
78.443
77.340
77.086
68.079
60.402
59.596
56.926
56.424
55.576
55.506
52.792
52.583
52.429
52.290
30.414
29.809
21.485
19.640
19.486
17.371
15.535
150 100 50 0
ppm (t1)

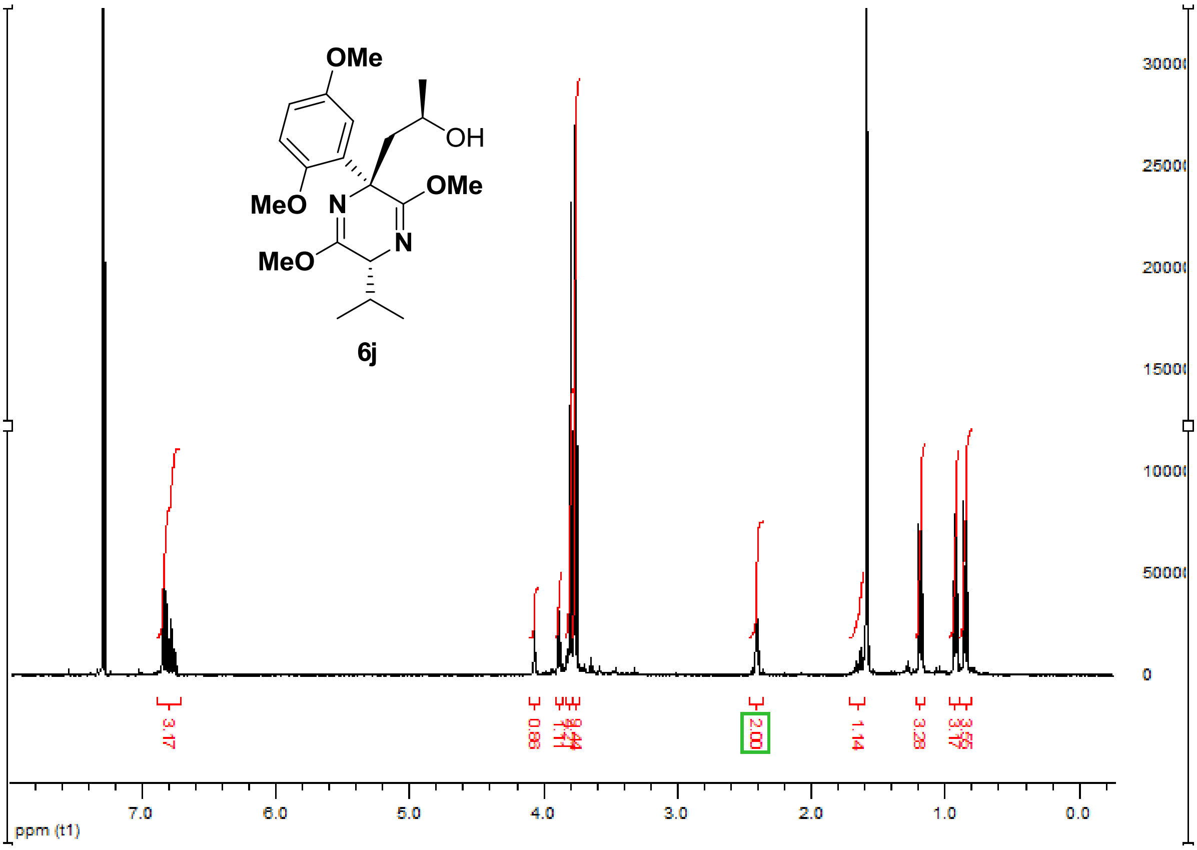
OMe
OH
MeO
N
OMe
N
MeO
6j
3.17
0.88
2.00
1.14
3.28
7.0
6.0
5.0
4.0
3.0
2.0
1.0
0.0
ppm (t1)
0

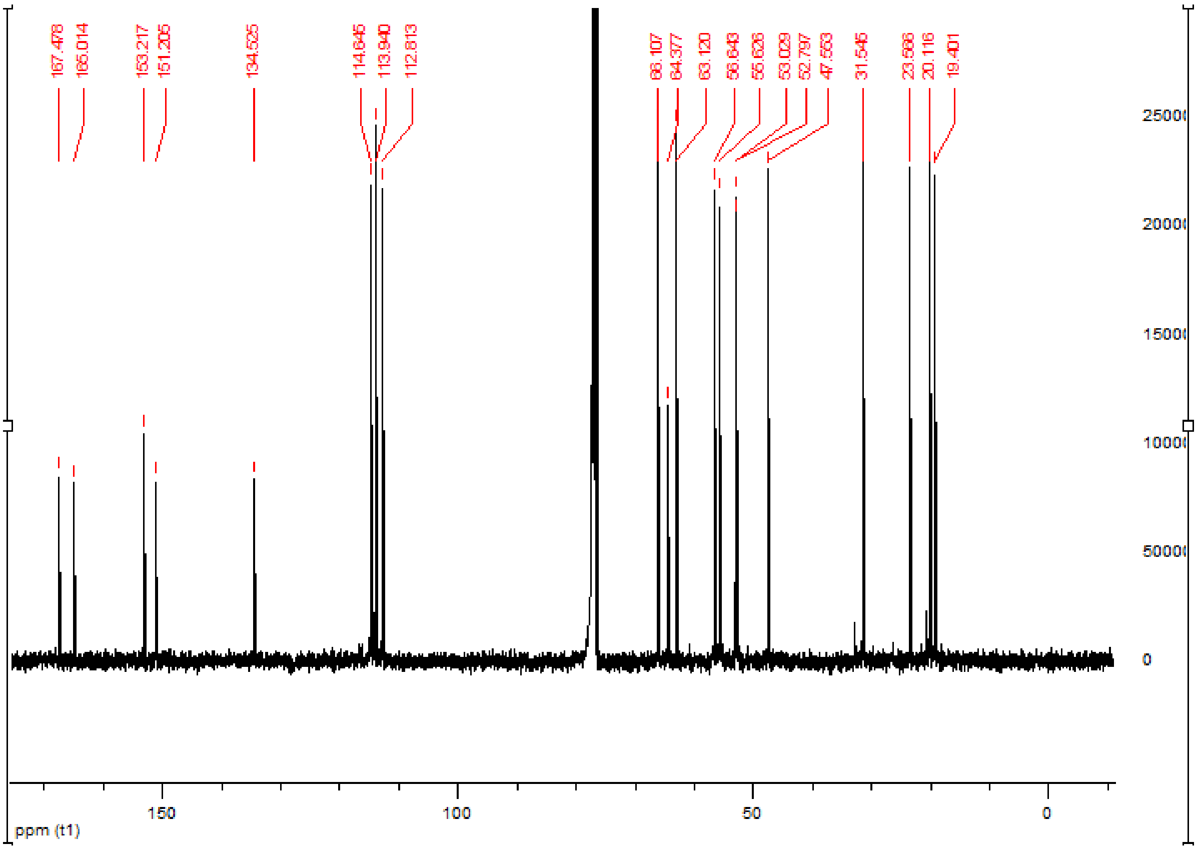
167.478
165.014
153.217
151.205
134.525
114.645
113.940
112.813
68.107
64.377
63.120
56.643
55.626
53.029
52.797
47.553
31.545
23.586
20.116
19.401
150
100
50
0
ppm (t1)
0

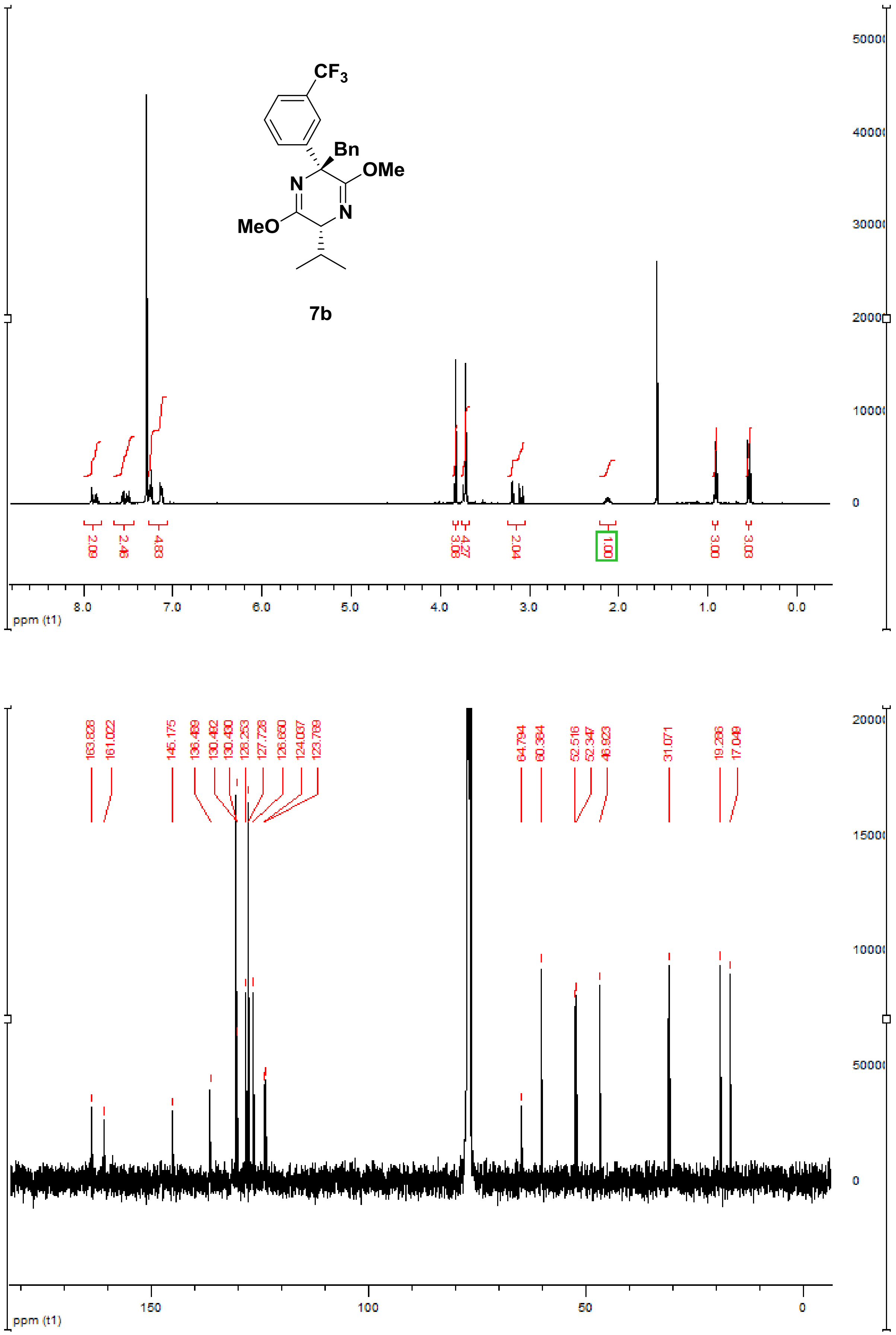
CF3
Bn
OMe
N
N
MeO
7b
2.09
2.46
4.83
3.06
4.27
2.04
1.00
3.00
3.03
ppm (t1)
163.828
161.022
145.175
136.499
130.462
130.430
128.253
127.728
126.860
124.037
123.789
64.794
60.394
52.516
52.347
46.923
31.071
19.286
17.049
ppm (t1)

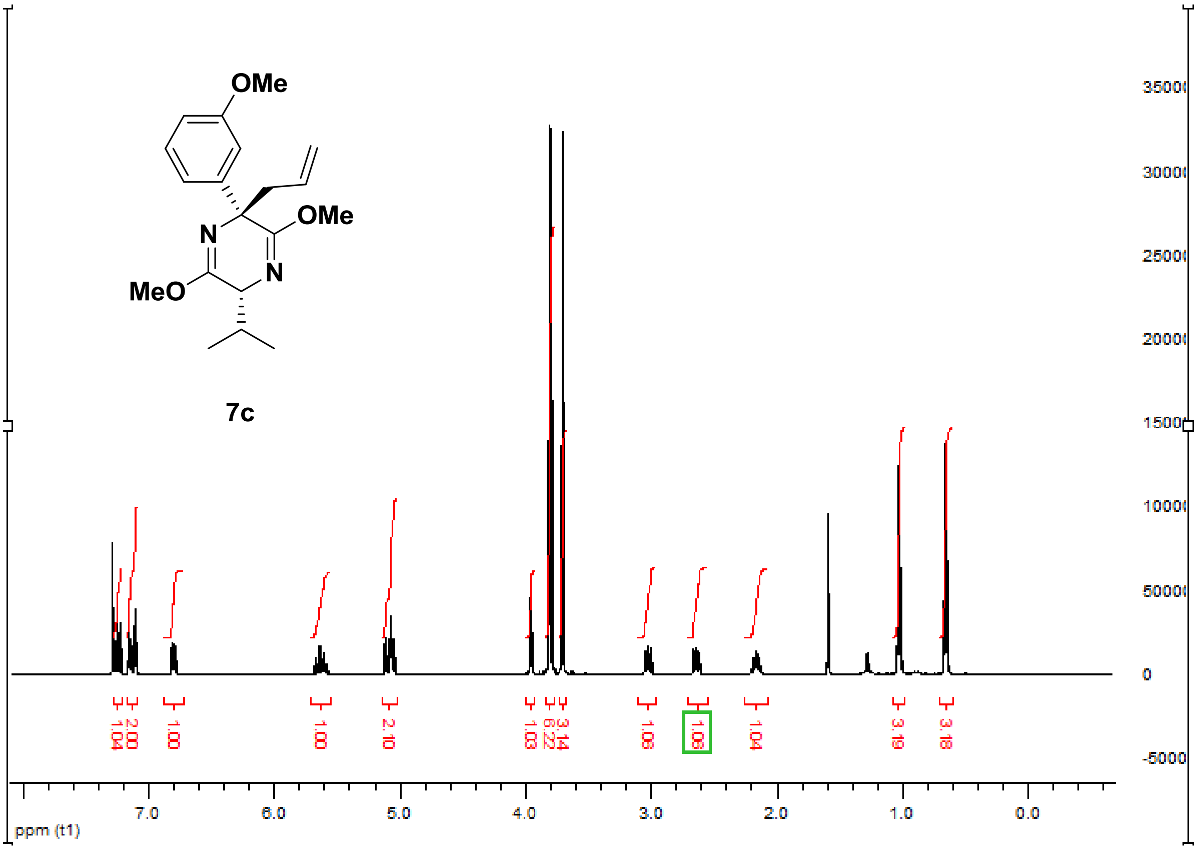
OMe
OMe
MeO
N
N
7c
1.04
2.00
1.00
1.00
2.10
1.03
6.22
3.14
1.06
1.06
1.04
3.19
3.18
ppm (t1)
7.0
6.0
5.0
4.0
3.0
2.0
1.0
0.0

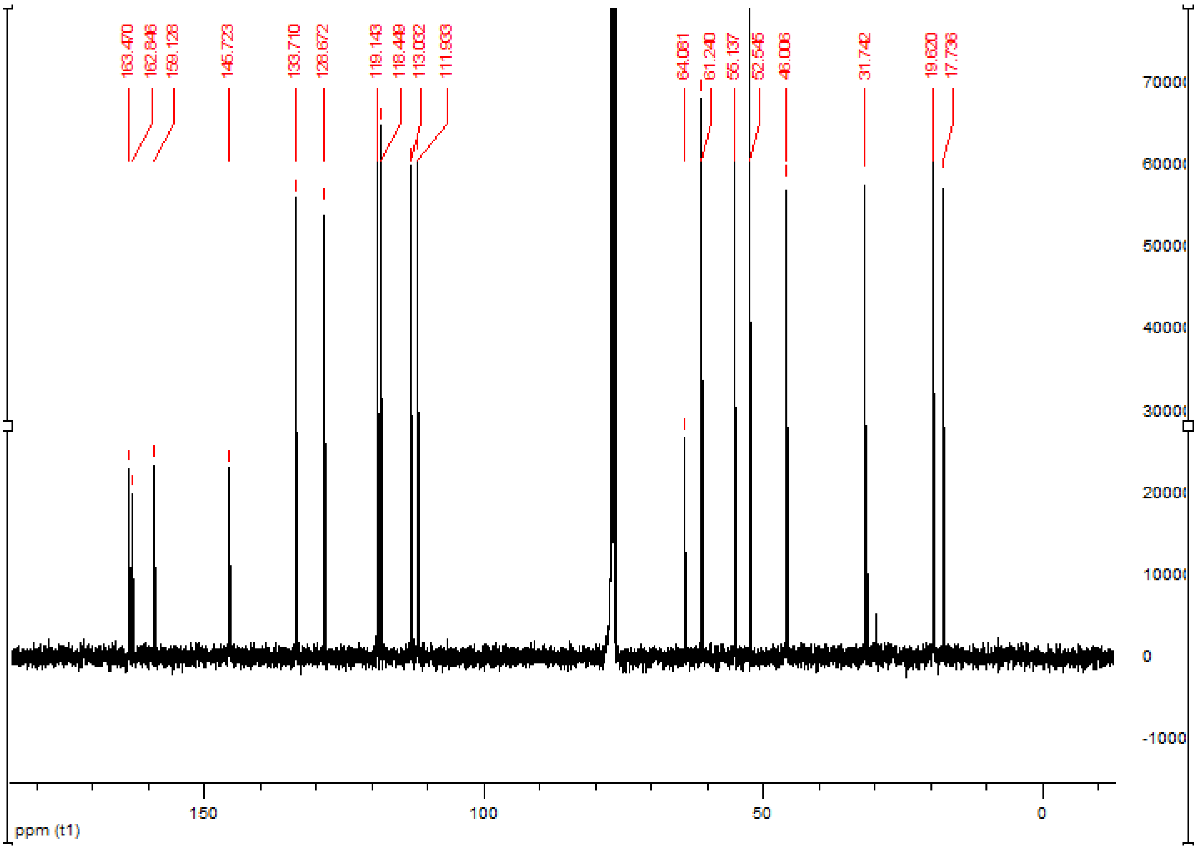
163.470
162.846
159.128
145.723
133.710
128.672
119.143
118.449
113.032
111.933
64.081
61.240
55.137
52.545
46.006
31.742
19.620
17.736
ppm (t1)
150
100
50
0
